# Structure and function of cancer-related developmentally regulated GTP-binding protein 1 (DRG1) is conserved between sponges and humans

**DOI:** 10.1038/s41598-022-15242-2

**Published:** 2022-07-05

**Authors:** Silvestar Beljan, Kristina Dominko, Antea Talajić, Andrea Hloušek-Kasun, Nikolina Škrobot Vidaček, Maja Herak Bosnar, Kristian Vlahoviček, Helena Ćetković

**Affiliations:** 1 Division of Molecular Biology, Ruđer Bošković Institute, 10000 Zagreb, Croatia; 2 Division of Biology, Faculty of Science, University of Zagreb, 10000 Zagreb, Croatia; 3 Division of Molecular Medicine, Ruđer Bošković Institute, 10000 Zagreb, Croatia

**Keywords:** DNA, Enzyme mechanisms, Proteins, RNA, Structural biology, Oncogenes, Cell migration, Cellular imaging, Cytoskeleton, Computational models, Phylogeny, Protein analysis, Protein folding, Protein structure predictions, Statistical methods, Evolutionary genetics, Molecular evolution, Evolutionary biology, Biochemistry, Cancer, Cell biology, Computational biology and bioinformatics, Evolution, Genetics

## Abstract

Cancer is a disease caused by errors within the multicellular system and it represents a major health issue in multicellular organisms. Although cancer research has advanced substantially, new approaches focusing on fundamental aspects of cancer origin and mechanisms of spreading are necessary. Comparative genomic studies have shown that most genes linked to human cancer emerged during the early evolution of Metazoa. Thus, basal animals without true tissues and organs, such as sponges (Porifera), might be an innovative model system for understanding the molecular mechanisms of proteins involved in cancer biology. One of these proteins is developmentally regulated GTP-binding protein 1 (DRG1), a GTPase stabilized by interaction with DRG family regulatory protein 1 (DFRP1). This study reveals a high evolutionary conservation of DRG1 gene/protein in metazoans. Our biochemical analysis and structural predictions show that both recombinant sponge and human DRG1 are predominantly monomers that form complexes with DFRP1 and bind non-specifically to RNA and DNA. We demonstrate the conservation of sponge and human DRG1 biological features, including intracellular localization and DRG1:DFRP1 binding, function of DRG1 in α-tubulin dynamics, and its role in cancer biology demonstrated by increased proliferation, migration and colonization in human cancer cells. These results suggest that the ancestor of all Metazoa already possessed DRG1 that is structurally and functionally similar to the human DRG1, even before the development of real tissues or tumors, indicating an important function of DRG1 in fundamental cellular pathways.

## Introduction

The developmentally regulated GTP-binding protein (DRG) subfamily is a member of the Obg family of GTPases^1^. The DRG subfamily contains relatively underexplored GTPases found in eukaryotes and archaea but not in bacteria. While archaea have only one DRG homolog^2,3^, most eukaryotes have two DRG homologs – DRG1 and DRG2. Three *drg* genes are present in some plants due to a lineage-specific duplication of the *drg2* gene^3,4^. All DRG proteins are highly similar in primary structure, suggesting their important roles in biological processes^2^. DRG1 and DRG2 are involved in protein translation, microtubule regulation, and cell growth^3^. In addition, DRG1 and DRG2 each have an evolutionarily conserved binding partner, the DRG family regulatory protein 1 and 2 (DFRP1 and DFRP2)^3,5^. Despite the high similarity between the human DRG1 and DRG2 (57% identity)^2^, DFRP1 and DFRP2 are strikingly different^5^ suggesting that the emergence of DFRPs contributed to the functional divergence of DRG1 and DRG2^6^.

Much more is known about DRG1 than DRG2, including its structure, regulation of GTPase activity, DFRP1 binding, cellular functions, and its association with disease^3^. DRG1 contains the N-terminal HTH domain, the canonical GTP-binding domain (G-domain) with five characteristic motifs (G1-G5), the S5D2L insertion domain and the C-terminal TGS domain^7^. The G-domain is the only structural similarity shared between DRG1 and other known GTPases. It has a potassium-dependent intrinsic GTPase activity that does not require GTPase-activating proteins (GAPs) and guanine nucleotide exchange factors (GEFs). Human DRG1 is active under a wide range of pH values and temperature, with an optimum at pH 8 to 9 and 42 °C, which implies that DRG1 may be involved in the cell stress response^8^. The DRG1 binding partner, DFRP1 (LEREPO4/ZC3H15), is also a highly conserved protein. It contains two CCCH-type N-terminal zinc fingers and a C-terminal domain that interacts with DRG1^5,7,9^. DFRP1 stimulates DRG1 GTPase activity by increasing its affinity for potassium ions^8^. The association with DFRP1 prevents DRG1 from ubiquitination and degradation by the proteasome machinery, and DFRP1 is considered as a stabilizing factor of DRG1. The downregulation of DFRP1 causes the downregulation of DRG1, while the overexpression of DRG1 by transient transfection is impossible without exogenous DFRP1^5^. Since DRG1 possesses the RNA binding activity and DRG1/DFRP1 co-sediments with polysomes, it is possible that they participate in the eukaryotic translation process or some other ribosome-related function^6,7,9,10^. Furthermore, DRG1 was identified as a microtubule-associated protein involved in microtubule bundling, polymerization and stabilization, and as a potential factor in chromatin decondensation^11^. These insights into the DRG1 functions indicate that DRG1 has a role in cell proliferation. Abnormal cellular proliferation is one of the hallmarks of cancer. However, the possible relationship between DRG1 and tumor initiation and progression remains unexplained. To this date, DRG1 has been assigned an oncogenic role in melanoma^12^, and DRG1 levels are increased in lung adenocarcinomas^13^. Further, different tumor cell lines exhibit high levels of DRG1 mRNA^2^. Downregulation of DRG1 in HeLa cells indicates that the protein is involved in mitotic spindle assembly^11^. Knockdown of DRG1 causes growth inhibition in M phase of HeLa, A549, and H1299 tumor cells, and vice versa, the overexpression of DRG1 leads to chromosomal missagregation^13^. Beside through its role in mitosis, DRG1 could be involved in cancer biology due to its additional function(s). For example, DRG1 can interact with other cancer-related proteins, such as c-myc, ras, and SCL/TAL1^14,15^. Considering all the available data, the exact biological function of DRG1/DFRP1 and its role in the most common diseases such as cancer, are not yet fully understood.

In recent years, there has been a growing interest in studying genes associated with cancer from an evolutionary perspective, as many of them appeared early in the evolution and are present in simple, nonbilaterian animals such as sponges (Porifera). Sponges are basal animals with simple morphology and a complex genome^16^. They consist of a few specialized cell types, lack true tissues and organs, and have numerous genes highly similar to their vertebrate homologs^17^. Many of those homologs have been implicated in the occurrence of tumors and tumor progression in humans. However, simple morphology and the lack of true tissues imply that the appearance of tumors in sponges is highly unlikely. Since sponges branched-off at the base of the animal tree, they are important for studying ancestral metazoan homologs before their diversification and specialization in complex "higher" animals, providing a new approach in understanding numerous biological processes, including cancer development and progression^16–20^. Earlier studies have shown that sponge proteins are highly similar in primary, predicted secondary, and tertiary structures, to homologs in "higher" metazoans, suggesting similar or identical biochemical and biological functions^17,18,21–29^. We have previously shown that at least some sponge proteins have biochemical and biological characteristics similar to their human homologs, including metastatic and/or tumor suppression properties^30–33^.

Due to high evolutionary conservation of the DRG1/DFRP1 complex, its link to basic cellular processes and role in cancer, research on DRG1 could significantly improve the understanding of these subjects. By using computational biology and a variety of biochemical and biological methods, we have unravelled the evolutionary history of DRG1 within metazoans and improved our knowledge on DRG1 features, including its structure, regulation and function.

## Results

### Distribution of the DRG protein subfamily across all domains of life and phylogenetic analyses

According to the publicly available genomic data, sponge *Amphimedon queenslandica* possesses two *drg* genes, *drg1* and *drg2*. For this study, we searched our unpublished transcriptomic data of the sponge *Eunapius subterraneus* and also found two proteins that belong to DRG subfamily. One of them encodes a 366 amino acid long typical DRG1 protein with conserved domains and active sites. According to the accepted nomenclature, the protein encoded by this gene is named DRG1. The other gene encodes the 364 amino acid long DRG2 protein with conserved domains and active sites. The protein encoded by this gene is named DRG2. We aligned the amino acid sequences of DRG1 proteins from animals and their closest unicellular relatives (a choanoflagellate and a filasterean) (Supplementary Fig. S1) and analysed the presence of the characteristic domains in the DRG1 homologs. The five G-motifs and two switch regions (Switch I and Switch II) important for GTPase activity are highly conserved from sponges to humans. HTH, S5DL2 and TGS domains are present in all metazoans and protists DRG1 homologs included in the analysis.

**Figure 1:**
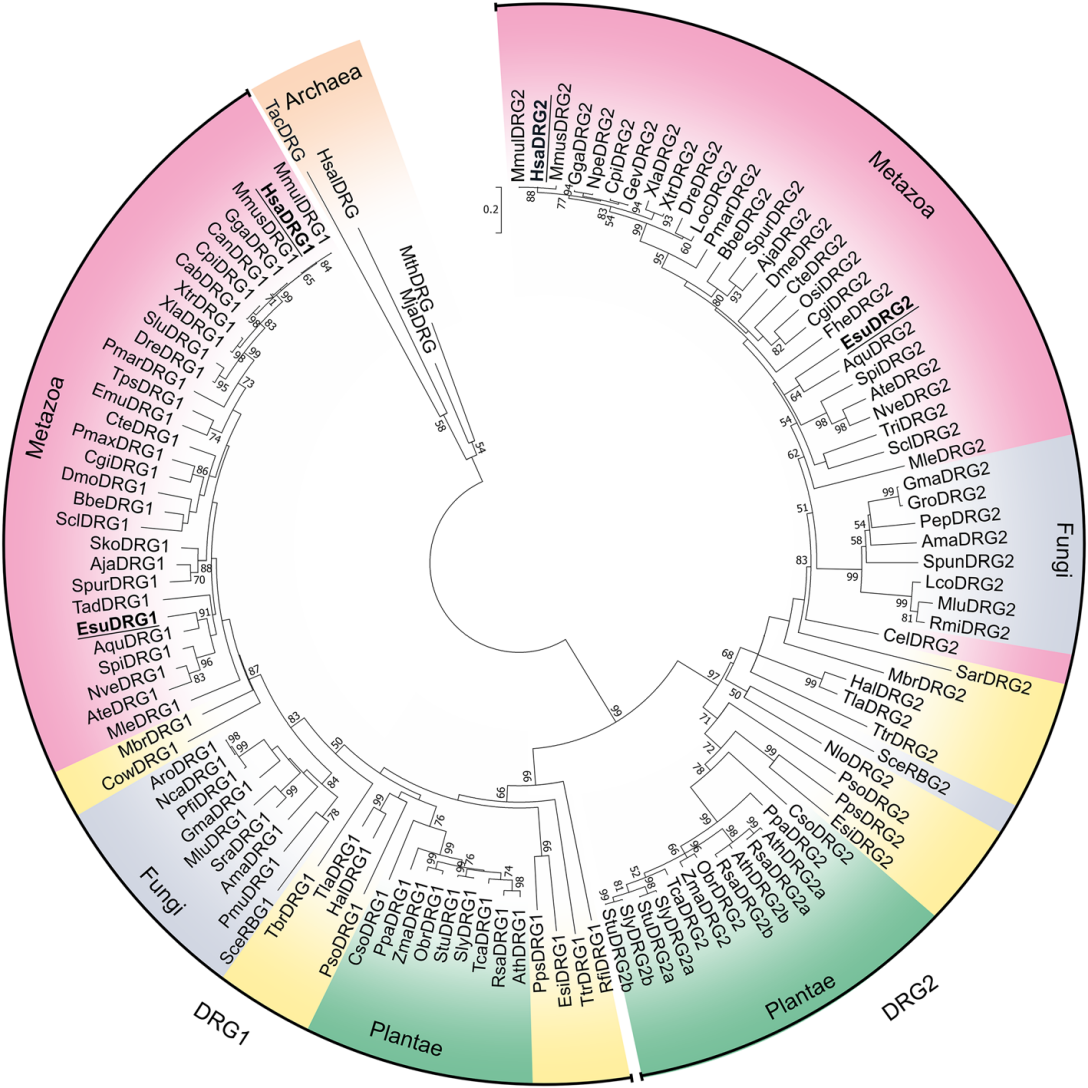
Phylogenetic relationships of DRG subfamily proteins in archaea and eukaryotes. The phylogenetic tree was rooted with archaeal DRG proteins as the outgroup. The numbers associated with branches are ML bootstrap values based on 1000 bootstrapping replications (bootstrap values higher than 50% are shown at the branching points). The scale bar indicates the number of substitutions per site. Accession numbers of the 122 amino acid sequences involved are listed in Supplementary Table S2. Taxonomic groups are highlighted in the following colors: Metazoa in pink, Fungi in grey, Plantae in green, Protista in yellow and Archaea in orange.

**Figure 2:**
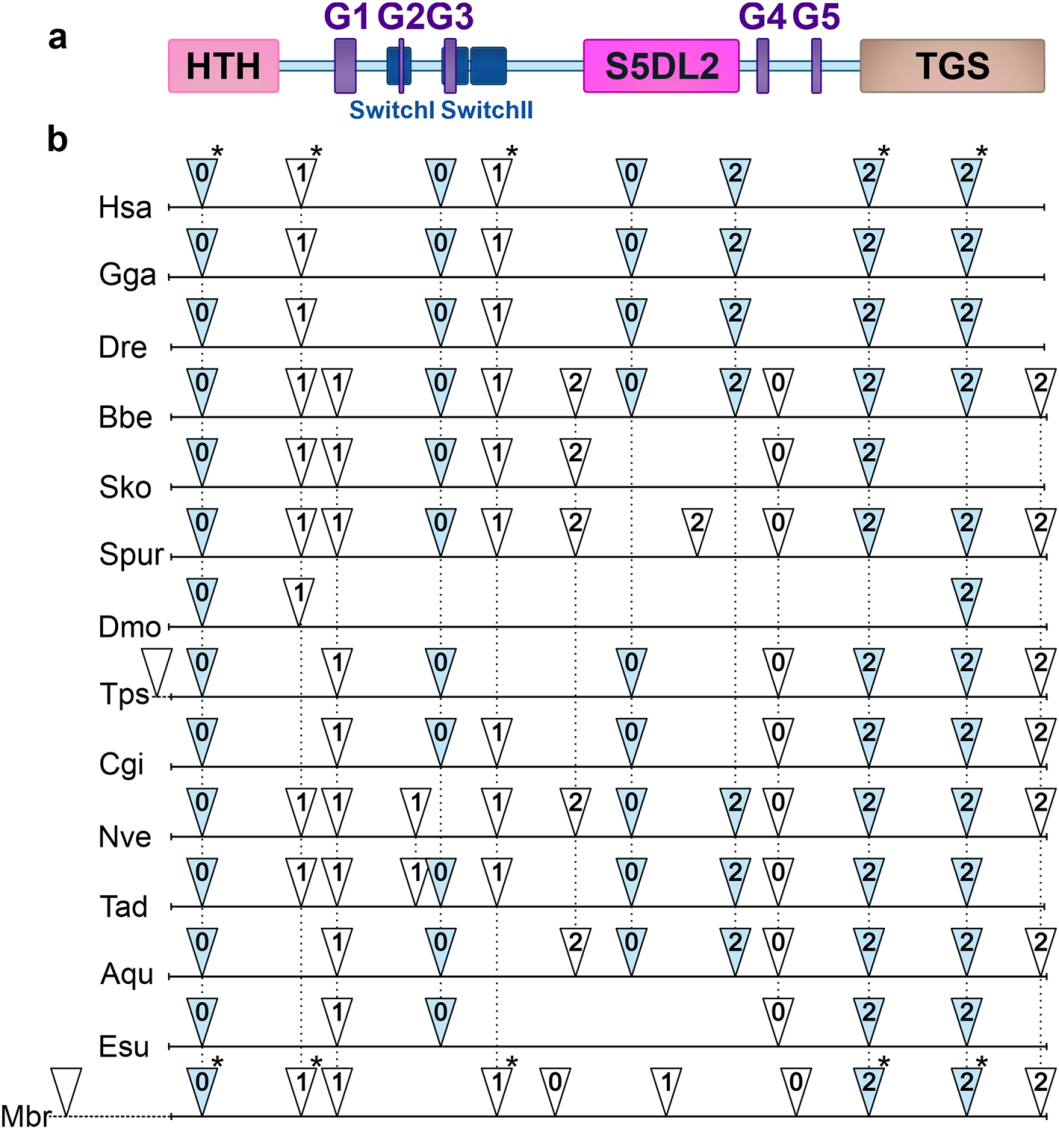
A schematic representation of human DRG1 protein and the positions of introns within *drg1* genes. (**a**) DRG1 protein consists of HTH (light pink), S5DL2 (magenta) and TGS (beige) domain, five G-domain motifs (purple) and two switch regions (dark blue). (**b**) Scheme of *drg1* genes from the representatives of metazoans and choanoflagellate showing intron positions. The positions of introns are marked by triangles and the number within the triangle denotes the intron phase. Introns that are in the same positions and phases based on the alignment of amino acid sequences are represented by black dashed lines. Introns present in human *drg1* gene and choanoflagellate homolog are marked with an asterisk, and those conserved from sponges to human are indicated by light blue color. Sequences of genes with indicated intron positions were taken from the NCBI's genomic database (https://www.ncbi.nlm.nih.gov/genome/).

To investigate the evolutionary history of DRG1, we have performed a comprehensive phylogenetic analysis of DRG family members that produced a well-supported tree (Fig. 1). We have demonstrated a deep branching between the DRG1 and the DRG2 protein groups (bootstrap values 99% and 97%, respectively), as all eukaryotic proteins clearly fell into one of these groups. Evolutionary relationships were generally well resolved, with defined clades for animals, fungi and plants (supported by high bootstrap values). Sponges, as expected, were placed at the base of the animal tree along with the representatives of other basal Metazoa (Cnidaria, Ctenophora and Placozoa). Our evolutionary analysis has shown that the DRG1 sequences of metazoans are closely related. They form a defined branch, with clades mostly corresponding to the taxonomic groups they belong to. DRG1 proteins from the close unicellular relatives of animals, *Monosiga brevicollis* and *Capsaspora owczarzaki*, formed a strongly supported sister groups with animals. DRG2 proteins also formed clades corresponding to taxonomy, but with a few exceptions. Among Chordata, *S. clava* homolog was placed next to the basal metazoan *T. adherans*, while *C. elegans* formed an independent branch, distant from other animals. Furthermore, *S. cerevisiae* RBG2 (DRG2) was more closely related to *Naegleria* DRG2 than to fungi, possibly due to a very early divergence. In contrast to DRG2, *S. cerevisiae* RBG1 (DRG1) is closely related to fungal homologs, as they formed a strongly supported branch (84% bootstrap value). Our study confirmed phylogenetic relationships for several protist lineages. Choanoflagellatea, Filasterea and Ichthyosporea formed a clade with animals and fungi as expected, because all these taxa belong to the eukaryotic supergroup Opisthokonta^34^. According to DRG2, amoeboid protists and apusomonads were shown to be related to Opisthokonta, which is consistent with their taxonomic position^35^. The positions of other protist supergroups included in the analysis were not robustly supported, possibly due to the incomplete genomic information, errors in sequencing, assembly, or annotation. Earlier studies have shown that *Arabidopsis* contains three DRG proteins^4,36^ and we found three DRGs in several other plant species. Two of the three plant DRG homologs are grouped with DRG2, which is probably a consequence of a lineage-specific duplication. We named these plant homologs DRG2a and DRG2b. The heatmap displaying multiple sequence alignments showed the highest homology for metazoan DRG1 proteins (75.7–100%). Although the DRG1 homology between kingdoms is lower, all DRG1 proteins showed identity/similarity higher than 50%, indicating evolutionary conservation of this protein in all eukaryotes (Supplementary Fig. S2). Sponge DRG1 proteins are highly homologous with the human DRG1, with 90.5–91.3% sequence similarity. We analysed intron–exon composition of the *drg1* genes from selected metazoans and a choanoflagellate to determine whether it follows the conservation pattern of the coding sequence. Our results have shown that only one intron, found in the HTH domain, is shared by all organisms analysed (Fig. 2). Two introns within the TGS domain are common for metazoan *drg1* genes and choanoflagellate homolog, and have identical intron phases. This indicates ancestral origin of both HTH and TGS protein domains. The human homolog has eight introns, six of which (both positions and phases) are conserved from sponge to human, with a few cases of intron loss in single species lineages. A major intron loss was observed in *Drosophila drg1* gene, suggesting accelerated evolution in this lineage^37^. Furthermore, we have analysed the sizes of introns throughout the metazoan evolution. The *drg1* homolog from sponge *A. queenslandica* contains 10 introns in the coding region, varying in size from 47 to 458 bp (data not shown). The *drg1* from sponge *E. subterraneus* contains only six relatively short introns. The human homolog has eight much longer introns, ranging from 277 to 9174 bp (data not shown). Although the lengths of the introns vary, multiple sequence alignment showed that positions and phases of six introns are conserved from sponges to humans.

### Sponge and human DRG1 show high structural similarity

**Figure 3:**
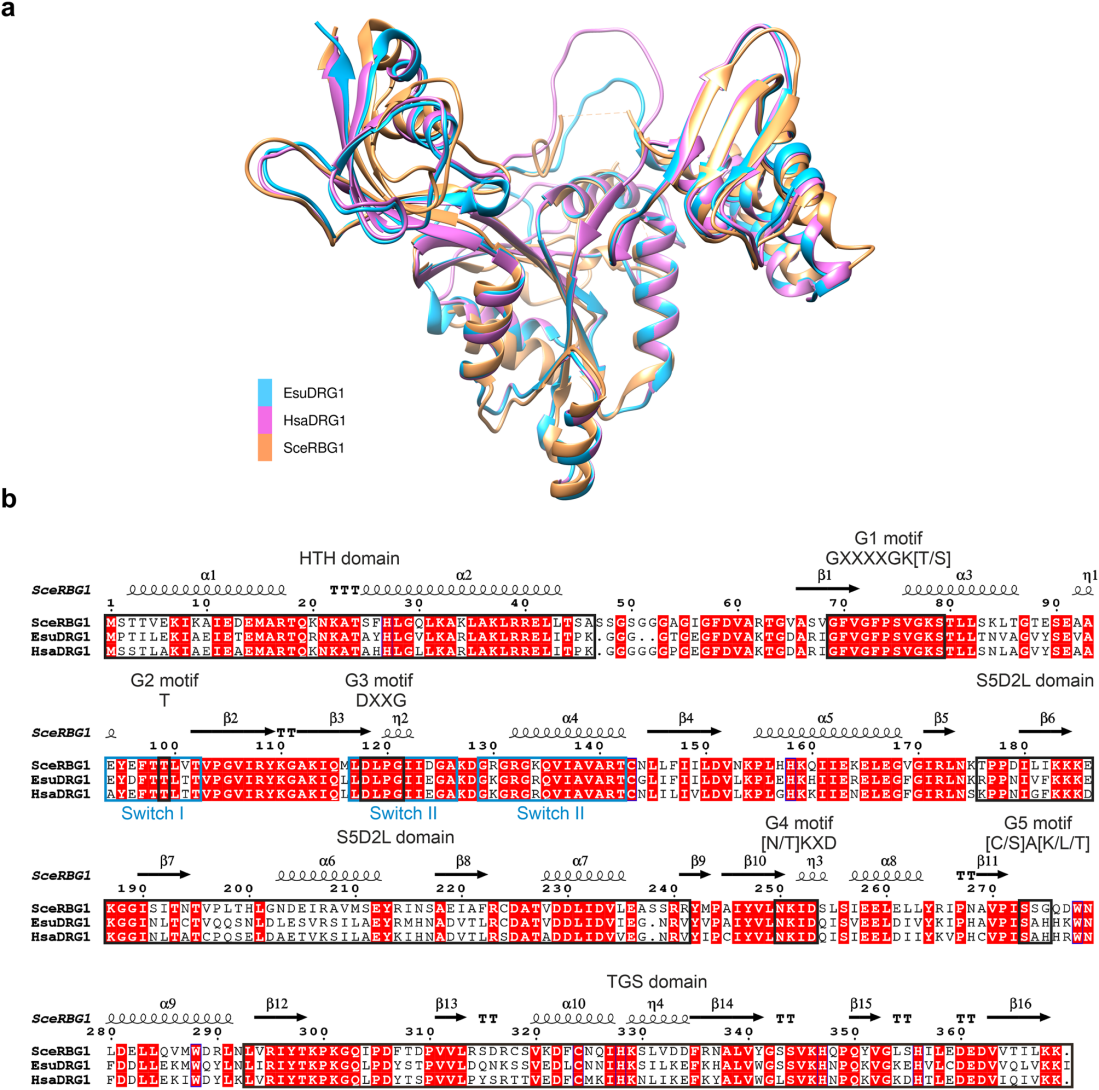
Sponge and human DRG1 show high structural similarity. (**a**) Superimposition of predicted DRG1 3D structures from *Eunapius subterraneus* (blue) and *Homo sapiens* (pink) with RBG1 crystal structure from *Saccharomyces cerevisiae* (PDB:4A9A) (orange). (**b**) Sequence alignment of DRG1 from *Eunapius subterraneus, Homo sapiens* and *Saccharomyces cerevisiae*. Regions of DRG1 proteins in boxes are indicated above the alignment, from N-terminus; HTH domain, G1 motif, G2 motif, G3 motif, S5D2L domain, G4 motif, G5 motif and TGS domain. The amino acid sequences of DRG1 were aligned using the Clustal Omega (https://www.ebi.ac.uk/Tools/msa/clustalo/); the secondary structures are predicted using the ESPript (http://espript.ibcp.fr/ESPript/ESPript/index.php). Red boxes with white letters indicate a strict identity.

In order to investigate structural similarity and conservation among the sponge, human and yeast DRG1 proteins, structures of EsuDRG1 and HsaDRG1 were predicted using the AlphaFold software^38^. Predicted models show high confidence in all regions except in some flexible loops (Supplementary Fig. S3). Predicted models superimpose very well with the yeast DRG1 protein, SceRBG1 (Fig. 3a). Previously determined 3D structure of the yeast SceRBG1 (PDB:4A9A) shows that it contains the N-terminal HTH domain, the G-domain with five characteristic motifs (G1-G5), the S5D2L insertion domain and the C-terminal TGS domain^7^. EsuDRG1 shows more similarity to the HsaDRG1 (RMSD: 0.660 Å, sequence identity = 80.7%) than to SceRBG1 (RMSD: 1.956 Å, sequence identity = 66.2%). The sequence alignment based on the resolved structure of SceRBG1 in Fig. 3b shows a high similarity and conservation of the main protein motifs and domains among these three DRG1 homologs. The main difference can be found in the conserved G-binding domain (G5 motif) where ^274^Ser-His-Gln in SceRBG1 is replaced with the Ala-His-His sequence in human and sponge homologs.

### Sponge DRG1 protein is a monomer and forms a complex with DFRP1

**Figure 4:**
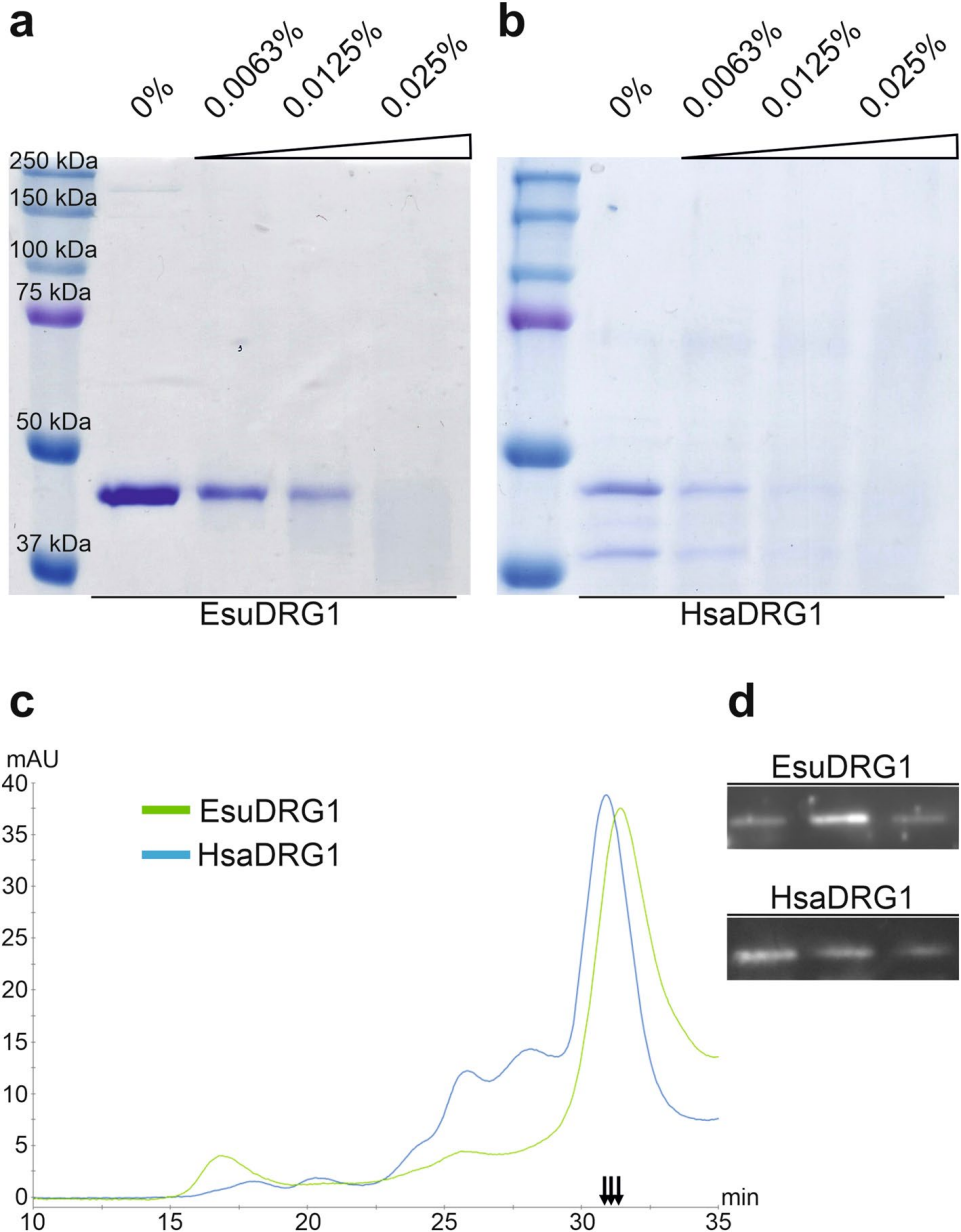
The sponge and human DRG1 proteins predominantly exist in the monomeric state. (**a**) Crosslinking of EsuDRG1 and (**b**) HsaDRG1. Glutaraldehyde was added up to a final amount of 0.025%. Reactions were incubated at 25 °C for 5 min. (**c**) SEC was performed by loading purified recombinant proteins EsuDRG1 (green line) and HsaDRG1 (blue line) onto a Superdex 200 Increase 10/300 GL column, calibrated for molecular mass analysis by a series of standard proteins. (**d**) Western blot analysis on selected fractions (indicated by arrows) with antibodies against His-tag. Original gels and blots are presented in Supplementary Fig. S8a,b.

**Figure 5:**
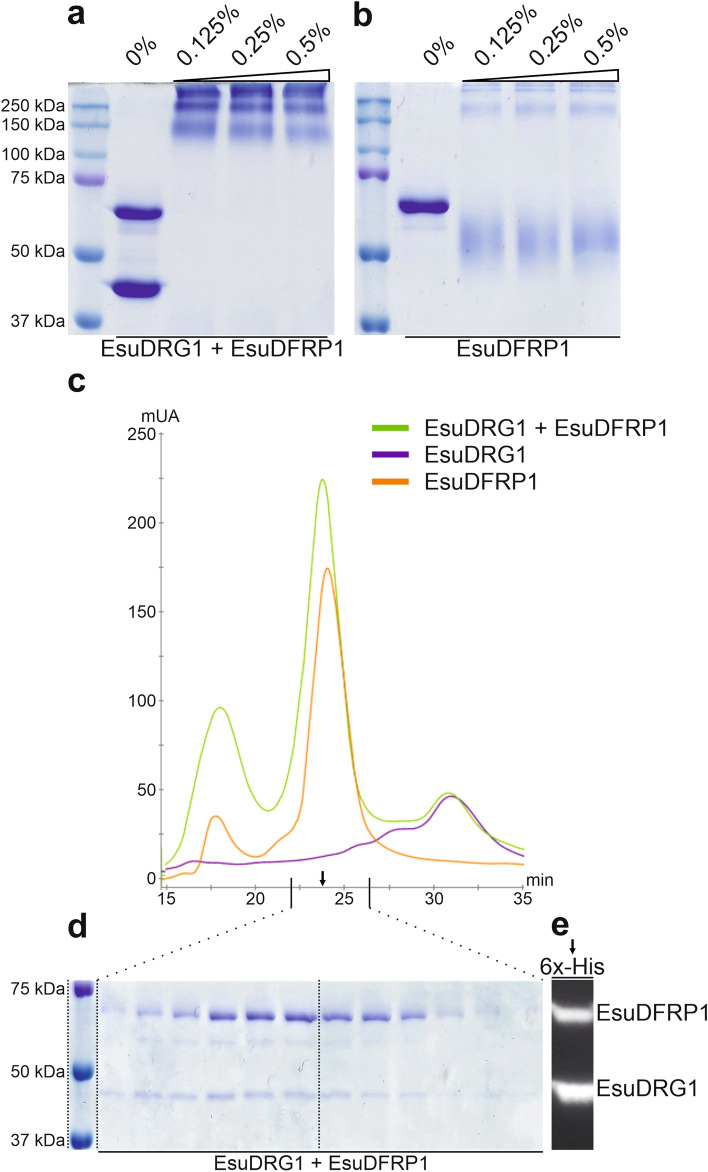
Sponge DRG1 and DFRP1 form heterooligomers. (**a**) Crosslinking of EsuDRG1 + EsuDFRP1 and (**b**) EsuDFRP1 alone. Glutaraldehyde was added up to a final amount of 0.5%. Reactions were incubated at 25 °C for 30 min. (**c**) SEC of EsuDRG1 protein (purple line), EsuDFRP1 protein (orange line) and a mixture of EsuDRG1 + EsuDFRP1 proteins (green line). (**d**) Selected fractions of potential DRG1 + DFRP1 complexes (at retention time 22–26 min) were analysed by Coomassie Brilliant Blue staining. (**e**) Western blot of selected fraction of EsuDRG1 + EsuDFRP1 (indicated by arrow) against His-tag. Original gels and blots are presented in Supplementary Fig. S8d,e.

To analyse the biochemical properties of EsuDRG1 protein, we produced and purified the recombinant EsuDRG1 protein and its binding partner EsuDFRP1. We also produced recombinant human DRG1 and DFRP1 proteins that served for comparison. First, we checked the oligomerization status of both EsuDRG1 and HsaDRG1 by crosslinking with glutaraldehyde. The recombinant EsuDRG1 protein appears to be predominantly in the monomeric form (Fig. 4a). The same was observed for recombinant HsaDRG1 (Fig. 4b). These results were verified by the size-exclusion chromatography (SEC). The chromatograms confirm that the EsuDRG1 protein is predominantly monomeric, as is the HsaDRG1 (Fig. 4c). Western blot analysis of selected fractions confirmed that the dominant peaks were indeed DRG1 protein (Fig. 4d). Next, we tested the presence of a DRG1 and DFRP1 complex by crosslinking. The SDS-PAGE analysis revealed only high molecular weight bands (> 130 kDa) with no visible monomeric form of DRG1, indicating that the EsuDRG1 indeed forms complexes with DFRP1 (Fig. 5a). A similar result was observed for a mixture of recombinant HsaDRG1 and HsaDFRP1 proteins (Supplementary Fig. S4). Since the glutaraldehyde test of the possible EsuDRG1:EsuDFRP1 interaction resulted in a very high molecular weight bands that correspond to a complex made of more than two monomers, we decided to employ SEC to get a more detailed insight in the oligomerization level. The eluted fractions were collected and analysed by SDS-PAGE and Western blot. The chromatogram shows a dominant peak at a retention time of 23.74 min, which corresponds to a molecular weight of 184 kDa or a DRG1:DFRP1 complex assembled of 4 to 5 monomers (Fig. 5c). SDS-PAGE analysis of the dominant peak (Fig. 5d) confirmed that the ratio of DRG1:DFRP1 in the obtained complex could be 1:3 or 1:4, but not 1:1 as it was previously assumed^8^. Additional Western blot analysis confirmed that these two bands were indeed DRG1 and DFRP1 eluted in the same fraction (Fig. 5e). To further clarify this result, we crosslinked EsuDFRP1 alone and showed that DFRP1 itself forms oligomers (Fig. 5b). In addition, SEC indicated that it exists predominantly as a tetramer (Fig. 5c). We also tried to test and compare HsaDRG1:HsaDFRP1 complex formation, but HsaDFRP1 could not be obtained in an adequate amount and quality. Instead, using SEC we examined a mixture of HsaDRG1 and EsuDFRP1, and obtained almost the same monomer ratio, 1:3 or 1:4 HsaDRG1:EsuDFRP1 in the formed complex (data not shown).

### The sponge DRG1 protein has an intrinsic GTPase activity enhanced by DFRP1

**Figure 6:**
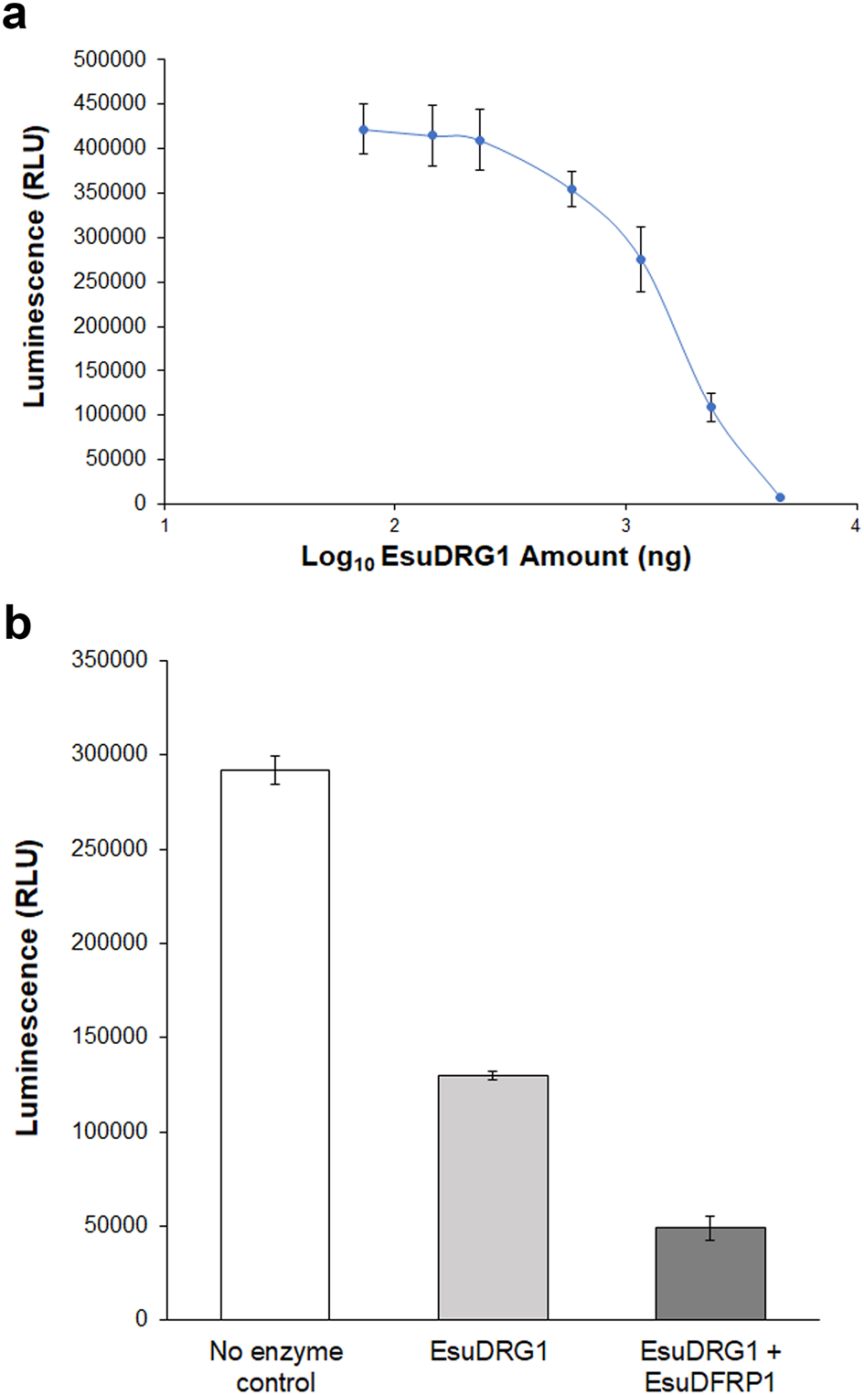
Intrinsic GTPase activity of the sponge DRG1 protein. (**a**) EsuDRG1 was serially diluted in the GTP/GAP buffer with fixed concentration of GTP (2 µM). Luminescence was measured after two hours. Standard deviations are indicated (mean ± SD, n = 3). RLU, relative luminescence unit. (**b**) DFRP1 promotes catalytic activity of the sponge protein DRG1. Concentration of each protein used in the reactions was 1.2 µM. The control sample contained only the GTP/GAP buffer. Standard deviations are indicated on the bars.

To test whether the EsuDRG1 has an intrinsic GTPase activity, we performed the luminescence-based GTP hydrolysis assays. In this assay, the generated luminescence signal correlates with the GTP concentration remaining in the reaction after GTP hydrolysis by GTPase. After incubating serial dilutions of EsuDRG1 protein with 2 µM GTP, we observed a decrease in luminescence signal correlating with an increase in EsuDRG1 concentration, confirming that EsuDRG1 protein has an intrinsic GTPase activity (Fig. 6a). EsuDRG1 shows very similar activity as HsaDRG1 at the same concentration (Supplementary Fig. S5). Moreover, the catalytic activity of EsuDRG1 is significantly higher (~ 2.5-fold) in the presence of its partner, DFRP1, at equimolar concentrations (Fig. 6b).

**Figure 7:**
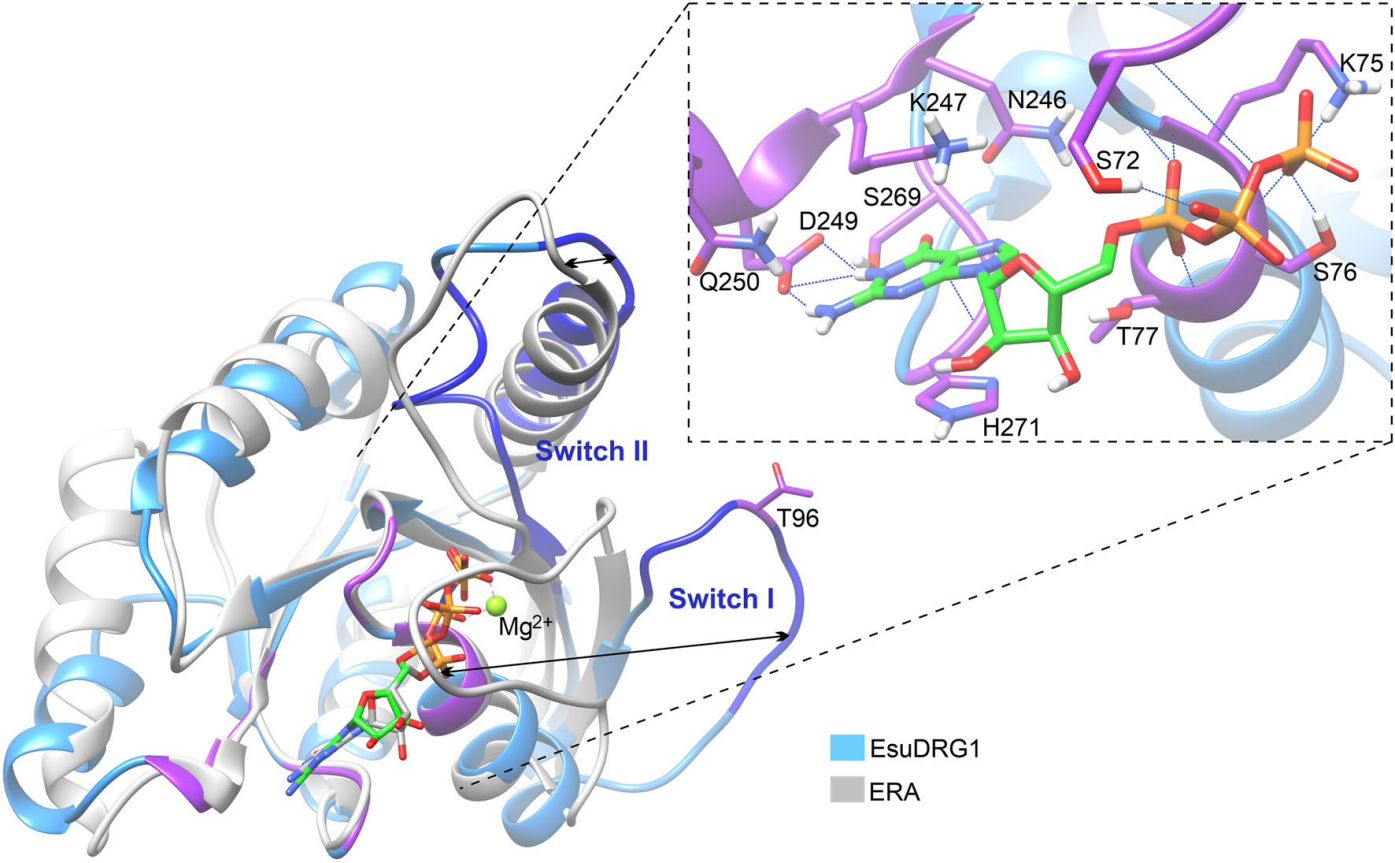
The superimposition of GTP-binding domains from *Eunapius subterraneus* DRG1 and *Aquifex aeolicus* ERA protein. Amino acids that are within 4 Å from GTP and highly conserved in the GTPase domain are shown as sticks, and colored purple. Different positions of Switch I and II are represented by arrows. GTP and its analogue are depicted as sticks. GTP docked into the DRG1 binding site using the AutoDock Vina is colored green. Zoomed GTP binding site of DRG1 from *Eunapius subterraneus* is depicted in the right rectangle. ERA protein from *Aquifex aeolicus* is crystalized in complex with MgGNP (PDB: 3IEV).

To get a better insight into the molecular mechanism of the DRG1s GTP binding, we employed several molecular modeling methods. First, to explore a GTP-binding site, a three-dimensional structure homology search was performed by using the DALI server and EsuDRG1 predicted model (http://www.ebi.ac.uk/dali). The DALI search returned several proteins with similar GTPase domains, including the *Aquifex aeolicus* ERA protein in complex with MgGNP (PDB: 3IEV). Structural alignment of the GTPase domains from *E. subterraneus* and ERA protein gave an RMSD value of 1.789 Å (Q-score: 0.113) across 130 aa pairs. The structure superimposition showed a well conserved GTP binding site. In order to further investigate the EsuDRG1 GTP binding mode, a docking calculation using AutoDock Vina^39^ was conducted. As a docking target previously defined GTP-binding site was selected. Conserved amino acids that are predicted to be involved in GTP binding were set as flexible during the docking. The docking result with the best docking score of -8.8 kcal/mol (Fig. 7) confirmed the characteristic GTP binding pattern carried out by the characteristic G-domain motifs^7^: P71, S72 and T77 within the G1 motif (GxxxxGK(S/T) make H-bonds with α- and β-phosphate groups of GTP. K247 and D249 from G4 [(N/T)KxD] motif establish H-bonds with guanine amine groups, while S269 from G5 motif ensures base discrimination as the H-bond acceptor that interacts with the guanine amino group. H271 from G5 motif makes a π–π stacking interaction that additionally ensure proper positioning of the guanine ring. G2 (Switch I, [x(T/S)x] and G3 (Switch II, [DxxG] that usually stabilize Mg^2+^ and γ-phosphate are in this case located a bit far away from the GTP. Indeed, as it can be seen in Fig. 7, the superimposition of EsuDRG1 and ERA:GTP complex shows a difference in Switch I, and II motif positions. The reason is that the Alpha fold predicted an open EsuDRG1 GTP-binding site conformation with Switch I and II displaced away from the GTP-binding site (Supplementary Fig. S3). The same relocation of Switch I and II motifs, as in ERA:GTP complex would probably happen in EsuDRG1 after productive GTP-binding. This relocation is characteristic for the GTPase domains^40^ and it has been shown that these conformational changes after GTP binding and hydrolysis transduce cellular signals to downstream effectors^41^.

### Sponge DRG1 binds RNA and DNA nonspecifically

**Figure 8:**
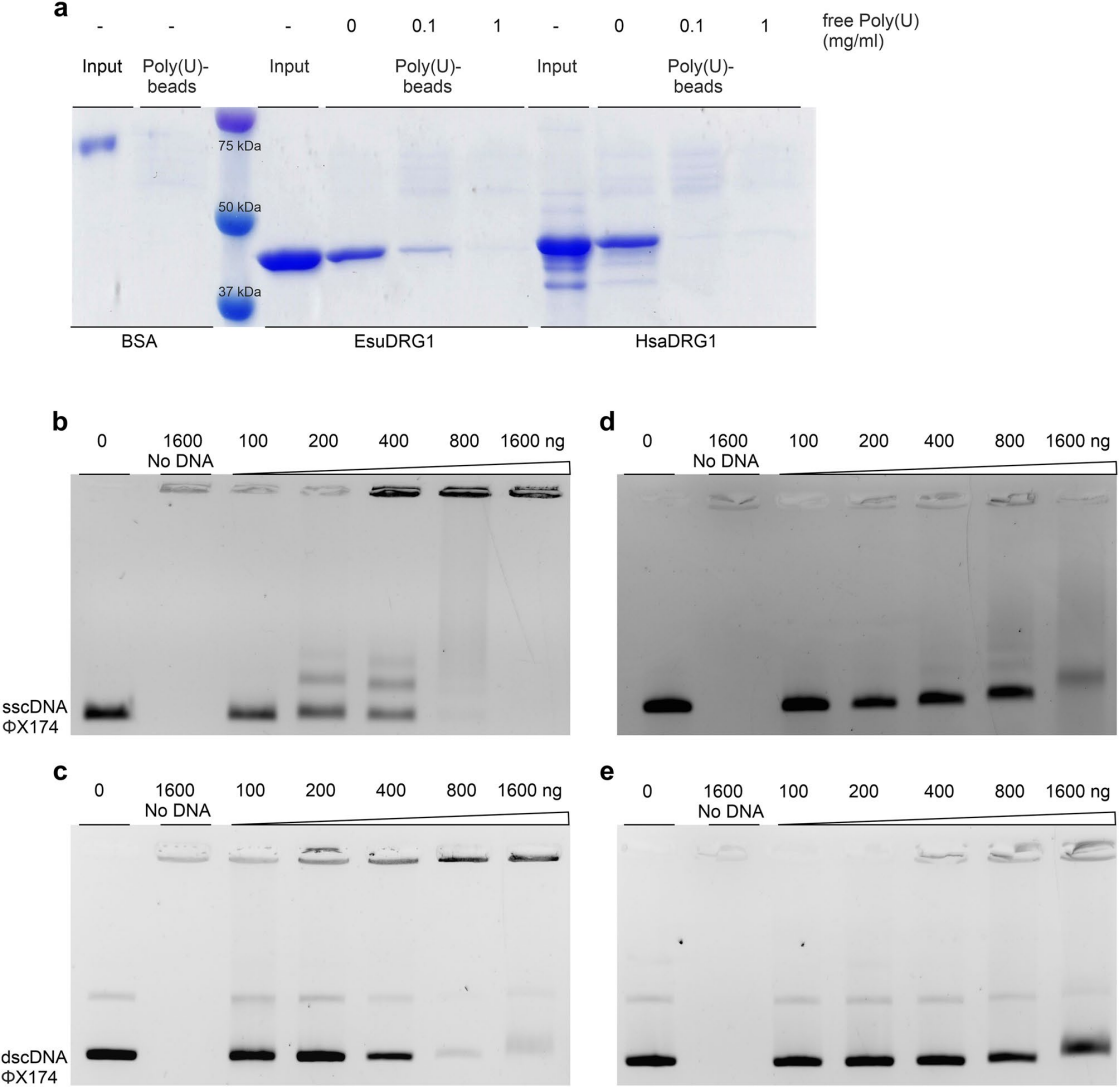
Nonspecific RNA and DNA binding activity of sponge protein DRG1. (**a**) Sponge and human protein DRG1 (500 ng) were preincubated with increased concentrations of free poly(U) followed by the incubation with 50% poly(U) agarose beads. BSA served as a negative control. After incubation, proteins were analysed by SDS-PAGE and stained with Coomassie Brilliant Blue. (**b**) Indicated amounts of purified EsuDRG1 protein (100–1600 ng) were assayed with 200 ng of single-stranded circular ΦX174 virion DNA or (**c**) double-stranded circular ΦX174 RFI DNA. Samples were analysed in a 0.5% agarose gel and stained with ethidium bromide. The same was repeated with purified human DRG1 protein, which served for comparison (**d**) and (**e**). Original gels are presented in Supplementary Fig. S8f.

Since it has been shown that DRG1 from yeast and *Xenopus laevis* can bind RNA^42,43^, we checked whether the EsuDRG1 has the same ability. To test the binding of DRG1 to RNA we used polyuridylic acid (poly(U)) agarose beads as described previously^43^. The results show that the EsuDRG1 bound to poly(U) agarose beads, whereas BSA, which served as a negative control, did not. To further analyse whether the binding is RNA specific, we used free poly(U) as a binding competitor. The incubation of DRG1 with an increased concentration of free poly(U) prior to the addition of poly(U) beads resulted in a dose-dependent decrease in binding. HsaDRG1, which we used for comparison, showed the same binding pattern (Fig. 8a). This result confirms that the EsuDRG1 protein alone has the ability to bind RNA. Since the EsuDRG1 has highly conserved S5D2L and TGS domains, typically found in RNA/DNA binding proteins, we also performed a DNA-binding shift assay. We have shown that purified EsuDRG1 and HsaDRG1 bind nonspecifically to the single-stranded circular DNA (sscDNA) and double-stranded circular DNA (dscDNA). Increasing amounts of EsuDRG1 or HsaDRG1 with a fixed amount of DNA alters DNA mobility, indicating the formation of protein-DNA complexes. The effect of EsuDRG1 binding to sscDNA is visible at 200 ng (Fig. 8b), while binding to dscDNA is visible at 800 ng of protein (Fig. 8c). EsuDRG1 amounts above 800 ng form large protein-sscDNA complexes that stack in wells (Fig. 8b). A similar effect was observed for HsaDRG1, but at higher protein amounts (Fig. 8d,e).

To gain insight into the binding of DNA and RNA to DRG1 on the molecular level, we used Haddock2.4 (https://wenmr.science.uu.nl/haddock2.4/) web server^44^. The best scored dsDNA, ssDNA and RNA positions are shown in Supplementary Fig. S6. All three nucleic acid molecules were placed into the electropositive groove formed at the contact of the S5DL2 and HTH domains of EsuDRG1.

### Biological characteristics of sponge and human DRG1 are conserved

**Figure 9:**
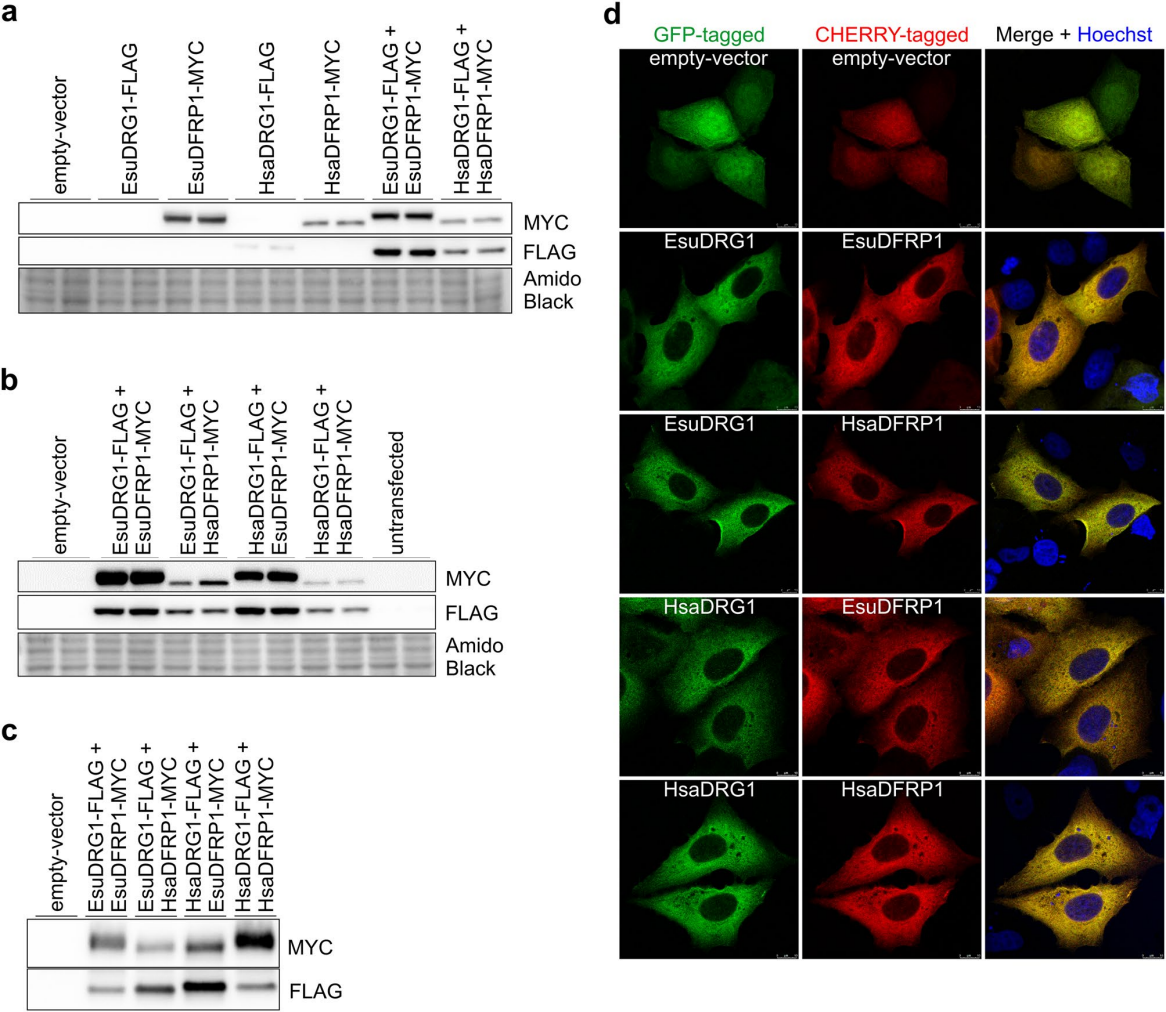
Biological characteristics of sponge and human DRG1 are similar. (**a**) Expression of DRG1 homolog from sponge *Eunapius subterraneus* is regulated by sponge DFRP1. (**b**) Levels of sponge homolog of DRG1 is regulated by either sponge or human DFRP1, and vice versa, human DRG1 is regulated by either sponge or human DFRP1*.* Levels of intra- and interspecies co-expressed sponge or human DRG1 and DFRP1 were analysed by Western blot and detected with antibodies against FLAG or MYC tag. Amido Black was used as a loading control. (**c**) In vivo interaction between DRG1 and DFRP1 is conserved from sponges to humans. DFRP1 was detected by Western blot analysis with antibody against MYC tag following the immunoprecipitation with antibody against FLAG tag. (**d**) Intracellular localization of both human DRG1 and its sponge homolog is in the cytosol. Colocalization (yellow) of human DRG1 or its sponge homolog with human or sponge DFRP1 in the cytosol of human breast cancer cells MCF-7. Human and sponge DRG1 were fluorescently labelled with GFP (green) and human and sponge DFRP1 with CHERRY (red). Hoechst was used to stain nuclei. The experiments were repeated three times in biological duplicates. Cells were analysed by confocal microscopy. Esu-sponge *Eunapius subterraneus*, Hsa-human. Cropped blots are displayed. Original blots are presented in Supplementary Fig. S8g,h,i.

To study the biological characteristics and functions of EsuDRG1 and HsaDRG1, as well as the EsuDFRP1 and HsaDFRP1, we have constructed mammalian expression vectors for transfection (Supplementary Table S1). We transfected MCF-7 human tumor cells with vectors with FLAG-tagged EsuDRG1 or HsaDRG1 and/or MYC-tagged EsuDFRP1 or HsaDFRP1 and analysed cell lysates by Western blot. While we detected high levels of exogenous EsuDFRP1 and HsaDFRP1, we did not detect exogenous EsuDRG1 nor HsaDRG1 if they were transfected without EsuDFRP1 or HsaDFRP1. However, when we co-transfected EsuDRG1 and EsuDFRP1, or HsaDRG1 and HsaDFRP1, we obtained high levels of both EsuDRG1 and HsaDRG1 in cell lysates (Fig. 9a). Based on these results, we assumed that EsuDRG1 needs to bind EsuDFRP1 for stabilization, similar to what is already known for HsaDRG1 and HsaDFRP1^5^. Next, we were interested if the binding between DRG1 and DFRP1 is conserved from sponges to humans. This was tested by co-transfecting EsuDRG1 and HsaDFRP1, and vice versa, HsaDRG1 and EsuDFRP1. We observed high levels of both EsuDRG1 or HsaDRG1 when exogenous DFRP1 was present, either EsuDFRP1 or HsaDFRP1 (Fig. 9b). These results indicate that the role of DFRP1 as a stabilizing factor that prevents ubiquitination and proteasomal degradation of DRG1 is conserved from sponges to humans. Our next aim was to confirm that the observed protein levels are due to direct binding of DRG1 and DFRP1. For that purpose, we co-transfected MCF-7 cells with FLAG-tagged EsuDRG1, or HsaDRG1 with MYC-tagged EsuDFRP1 or HsaDFRP1. The cell extracts were subjected to immunoprecipitation with anti-FLAG antibody. The precipitates were analysed by Western blot. In all precipitates, we detected exogenous DRG1 and DFRP1, confirming the conserved direct binding of DRG1 and DFRP1 from sponges to humans (Fig. 9c). Next, we analysed the intracellular localization of DRG1 in MCF-7 cells. Firstly, we confirmed that the endogenous DRG1 and DFRP1 are localized in the cytosol of human MCF-7 cells. Since both antibodies against DRG1 and DFRP1 were raised in the same animal species, we were not able to co-stain endogenous DRG1 and DFRP1. However, due to their localization we can presume complete co-localization of endogenous DRG1 and DFRP1 in the cytosol of MCF-7 cells (results not shown). To analyse the intracellular localization of EsuDRG1 and compare it with the HsaDRG1 localization, we constructed GFP-tagged vectors for transfection of MCF-7 cells (Supplementary Table S1). In addition, we constructed CHERRY-tagged vectors for EsuDFRP1 and HsaDFRP1. Since we could not detect a GFP signal after transfection of EsuDRG1 or HsaDRG1 alone, similar to our findings in cell lysates, we co-transfected EsuDRG1-GFP or HsaDRG1-GFP with EsuDFRP1-CHERRY or HsaDFRP1-CHERRY. In all four different combination of intra- or interspecies co-transfections of DRG1 and DFRP1, we observed complete co-localization of DRG1 and DFRP1 in the cytosol of MCF-7 cells and not in the nucleus (Fig. 9d). Identical localization of DRG1 and DFRP1 was observed in HeLa cells (Supplementary Fig. S7). Besides conserved binding of DRG1 and DFRP1, these findings also confirm the identical localization of EsuDRG1 and HsaDRG1, which may indicate their similar role(s) in human cancer cells.

### The role of DRG1 in α-tubulin dynamics is similar in sponges and humans

**Figure 10:**
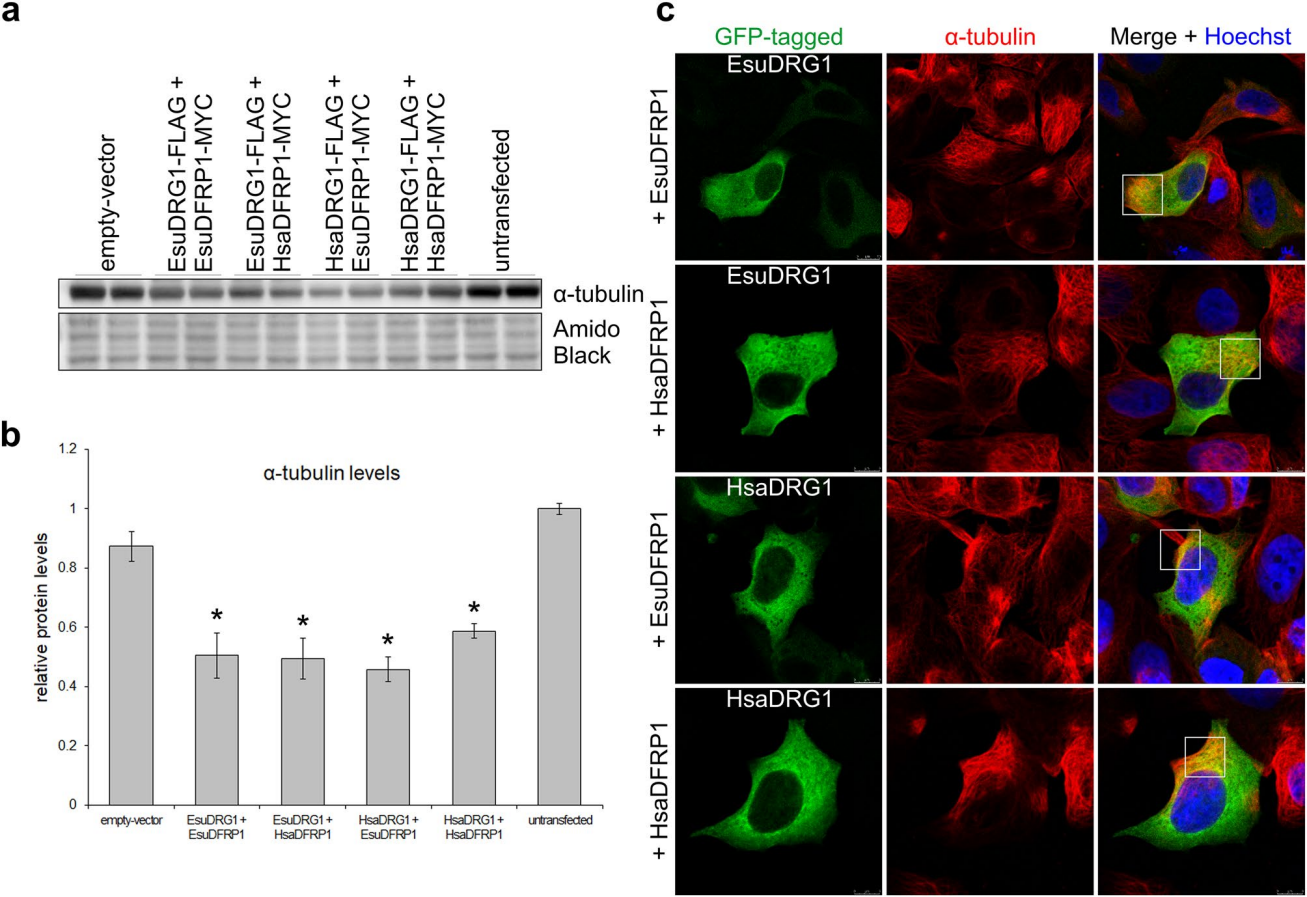
Sponge DRG1 co-localizes with α-tubulin and causes its decreased levels, similar to human DRG1. MCF-7 cells were co-transfected with human DRG1 or its sponge homolog (GFP-labelled) with human or sponge DFRP1 (not shown) and α-tubulin was detected with a specific antibody. (**a**) Western blot of α-tubulin levels. (**b**) Quantification of α-tubulin compared to empty-vector, *p < 0.05. (**c**) Colocalization (yellow square) of α-tubulin (red) with human and sponge DRG1-GFP (green). Hoechst was used to stain nuclei. The experiments were repeated three times in biological duplicates. Esu-sponge *Eunapius subterraneus*, Hsa-human. Original blots are presented in Supplementary Fig. S8j.

It was previously shown that HsaDRG1 has a role in binding, bundling, polymerization and stabilization of microtubules^11^. Therefore, our next aim was to study the biological function of the EsuDRG1 homolog in α-tubulin dynamics. For that purpose we co-transfected FLAG-tagged EsuDRG1 or HsaDRG1 with either MYC-tagged EsuDFRP1 or HsaDFRP1 and analysed the levels of α-tubulin (Fig. 10a). Our experiments show that the overexpression of EsuDRG1 caused a decrease in cellular α-tubulin levels compared to cells transfected with empty-vector, either when it was co-transfected with EsuDFRP1 (*p* = 0.0192) or HsaDFRP1 (*p* = 0.0386). In addition, lower levels of α-tubulin were statistically significant when we overexpressed HsaDRG1 with EsuDFRP1 or HsaDFRP1, *p* = 0.0371 and *p* = 0.0457, respectively (Fig. 10b). Since changes in α-tubulin levels due to over-expression of DRG1 were noticed, we analysed if there is a co-localization of EsuDRG1 and α-tubulin in MCF-7 (Fig. 10c) and HeLa cells (Supplementary Fig. S8). Once again, we co-transfected GFP-tagged EsuDRG1 or HsaDRG1 with CHERRY-tagged EsuDFRP1 or HsaDFRP1 after which we immunostained α-tubulin with a specific antibody. We observed co-localization of EsuDRG1 or HsaDRG1 with α-tubulin in the cell cytosol of all four co-transfected samples (Fig. 10c and Supplementary Fig. S8). These results, based on similar localization and changes in the levels of α-tubulin due to DRG1 over-expression, indicate a similar and conserved function of sponge and human DRG1 homolog in α-tubulin dynamics.

### Human DRG1 and its sponge homolog have similar functions in tumor biology

**Figure 11:**
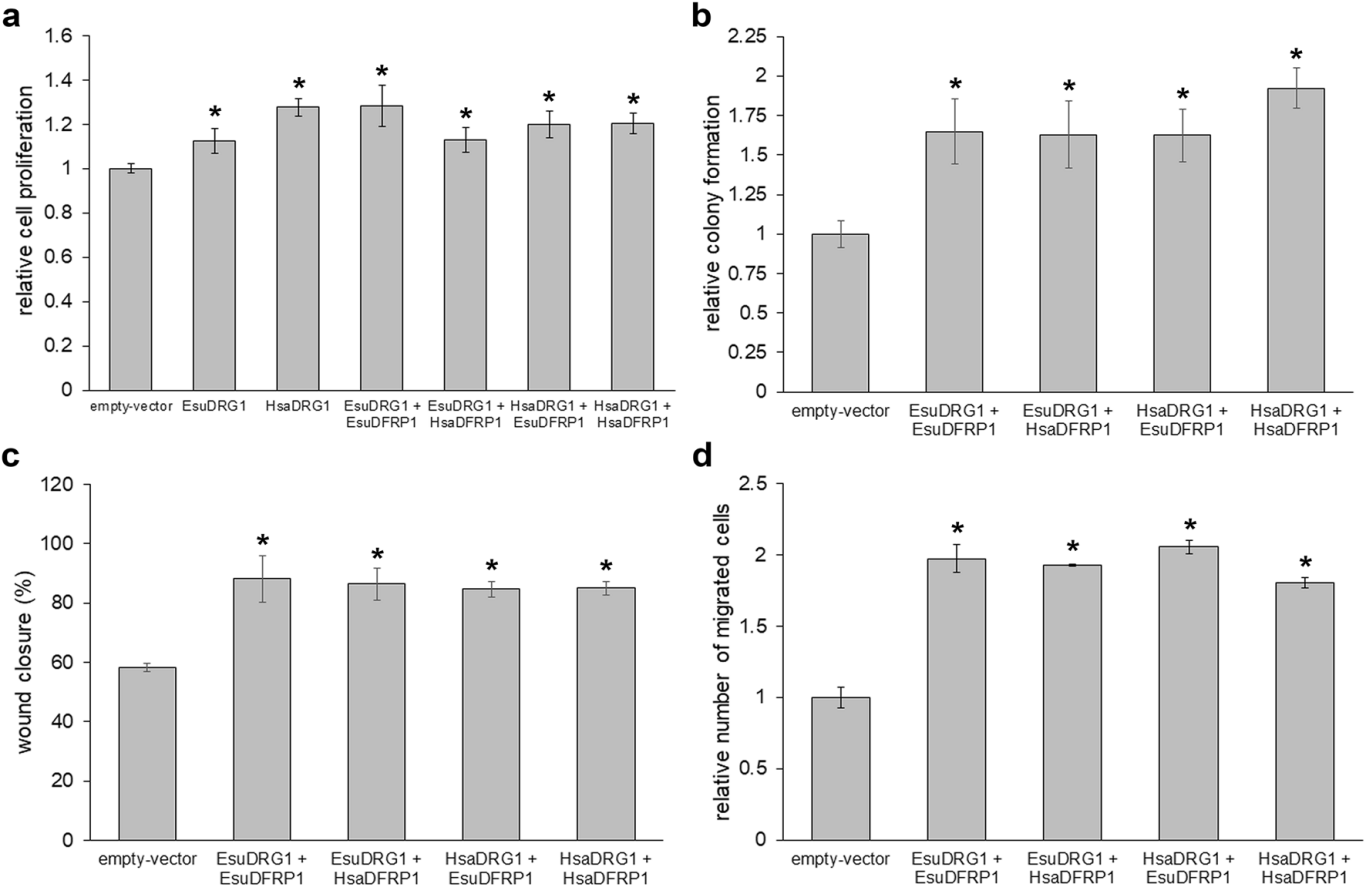
Both sponge and human DRG1 increase cell proliferation, colonization and migration of MDA-MB-231 cells. The function of sponge DRG1 is regulated by sponge or human DFRP1 in (**a**) cell proliferation, (**b**) colony formation, (**c**) wound healing and (**d**) cell migration in co-transfected MDA-MB-231 cells and compared to human DRG1. Quantification was done using the ImageJ software (National Institutes of Health, USA). The statistical significance of the tests was set at *p* < 0.05. The experiments were repeated three times in biological duplicates. Esu-sponge *Eunapius subterraneus*, Hsa-human. *p < 0.05.

Since several previous studies suggested that DRG1 and DFRP1 are required for growth, proliferation and migration of various cancer cell lines^13,45,46^,our next goal was to study the biological effect of EsuDRG1 on cancer cells. First, we analysed the role of EsuDRG1 in cell proliferation (Fig. 11a). All cells expressing exogenous EsuDRG1 or HsaDRG1 without DFRP1 exhibited increased cell proliferation compared to empty-vector as a control (EsuDRG1 (*p* = 0.0032), HsaDRG1 (*p* < 0.0001)). We observed a similar increase in cell proliferation when EsuDRG1 was co-transfected with EsuDFRP1 (*p* = 0.000374) or HsaDFRP1 (*p* = 0.002508), as well as when HsaDRG1 was co-transfected with EsuDFRP1 (*p* = 0.000284) or HsaDFRP1 (*p* < 0.0001) (Fig. 11a). These results show that the exogenous EsuDRG1 or HsaDRG1, although undetectable by other methods, are probably present and stabilised by endogenous DFRP1, which is sufficient to cause an increase in cell proliferation. Next, we examined the role of EsuDRG1 and HsaDRG1 in cell survival and colony formation. Once again, we co-transfected EsuDRG1 or HsaDRG1 with EsuDFRP1 or HsaDFRP1. Exogenous EsuDRG1 increased cell survival in combination with EsuDFRP1 (*p* = 0.006930) or HsaDFRP1 (*p* = 0.0088), and HsaDRG1 increased cell survival in combination with EsuDFRP1 (*p* = 0.0042) or HsaDFRP1 (*p* = 0.0004) (Fig. 11b). To test the role of EsuDRG1 and HsaDRG1 in cell migration, we used wound healing and Boyden chamber based assays (Fig. 11c,d). We observed enhanced cell migration and faster wound healing in all four co-transfected samples in comparison to control. Specifically, EsuDRG1 significantly increases the percentage of wound closure while co-expressed with EsuDFRP1 (*p* = 0.008) or HsaDFRP1 (*p* = 0.0137). A similar effect was observed for HsaDRG1 co-expressed with EsuDFRP1 (*p* = 0.0102) or HsaDFRP1 (*p* = 0.0091) (Fig. 11c). Boyden chamber based assay confirmed the increased cell migration after co-expression of EsuDRG1 or HsaDRG1 with EsuDFRP1 or HsaDFRP1 (*p* < 0.0001) compared to control (Fig. 11d). These results confirmed the conserved function of human DRG1 and its sponge homolog in tumor-related processes, including cell proliferation, colonization and migration.

## Discussion

The main objective of this study was to characterise a DRG1 protein from the sponge *Eunapius subterraneus* (EsuDRG1), in order to unravel the evolution of DRG1 and its functions, especially at the origin of animals. Here, we have performed the first comprehensive phylogenetic analysis of the DRG protein family, which includes representatives of all eukaryotic supergroups and archaea. To our knowledge, this type of analysis has not been performed before, although preliminary phylogenetic analyses have been published^3,47,48^. The strongly supported phylogenetic tree showed deep branching between DRG1 and DRG2, indicating divergence by gene duplication that probably occurred during the emergence of eukaryotes. Previously it was shown that *Arabidopsis* contains three DRG proteins, the homolog of human DRG1 and two DRGs that are 95% identical to each other^4,36^. We confirmed that the latter two proteins belong to DRG2 protein family, and we found three DRGs in some other plant species (e.g. *Raphanus*, *Solanum*). DRG2 proteins from plants (including green algae) were reported to be longer than the canonical DRG2 (365–370 amino acids) as a consequence of an extension at their C-termini^49^. We have found that some protists (the oomycete *Phytophthora*, the ciliate *Paramecium* and the brown alga *Ectocarpus*) also have the extensions at their C-termini. Our intron/exon structure analysis revealed that five introns found in the *drg1* genes of metazoans are most likely ancient, as they were also present in the choanoflagellate homolog, suggesting that the ancestral metazoan *drg1* gene was intron-rich. In addition, we confirmed that *drg1* genes from sponges have significantly shorter introns than the human homolog, while exon lengths are quite similar. The same has been previously documented for several other sponge genes^16,33,50^.

Biochemical characterization of the sponge and human DRG1 shows that they have identical properties. Glutaraldehyde crosslinking and gel filtration confirmed that both recombinant proteins are predominantly monomeric and form a complex with their binding partner, DFRP1. Moreover, we showed for the first time that the both EsuDRG1:EsuDFRP1 and HsaDRG1:EsuDFRP1 ratios in the stable complex are 1:3 or 1:4. Previous studies on the yeast DRG1 (RBG1):Tma46 and human DRG1:DFRP1 suggested a 1:1 ratio^7,8^. In addition, we showed that the stable DFRP1 protein is predominantly present in a form of a tetramer. The prediction of its 3D structure via Alpha fold resulted in a structure that is mostly composed of loops and disordered regions. This indicates that DFRP1 is an intrinsically disordered protein (IDP) same as DFRP2 (Supplementary Fig. S9)^7,51^. This is in accordance with low content of hydrophobic and aromatic residues (33.1%) and high percentage of polar and charged amino acids (66.9%) in DFRP1. This results in a large net charge of -26.374 at pH 7.4, characteristic for IDPs^52^. We presume that its structure becomes more ordered after oligomerisation and binding of proper interactors.

The sponge DRG1 possesses the intrinsic GTPase activity enhanced by DFRP1, same as the human homolog. Further, the docking calculation confirmed the characteristic GTP binding pattern carried out by the characteristic G-domain motifs. We confirmed that both sponge and human DRG1 also bind RNA nonspecifically. We have shown for the first time that the sponge and human DRG1 bind nonspecifically to single-stranded and double-stranded circular DNA. In addition, the Haddock prediction revealed a potential nucleic acid binding site that binds ds, ssDNA and RNA well. The binding site is positioned at the close contact of HTH and S5D2L domains that form an electropositive groove. This finding is in agreement with previous predictions^7^ and sequence analysis which showed that S5D2L domain, typically found in RNA/DNA binding proteins^53,54^, is well conserved in sponge and human DRG1 homologs. The role of DRG1 in DNA binding within a cell needs further investigation.

In order to test the biological function of sponge DRG1 and compare it to human DRG1, we analysed the function of exogenous sponge and human DRG1 in human tumor cells. We could not obtain viable cells after solely expressing the exogenous human DRG1 or sponge DRG1, without DFRP1, which is in accordance with other studies that have shown similar effects for human DRG1. However, we detected high levels of exogenous sponge DRG1 when co-transfected with DFRP1. We presume that this is a consequence of binding and stabilisation of the sponge DRG1 by DFRP1 which prevents its polyubiquitination and subsequent proteasomal degradation^5,15,55^, as already described for the human variant. In addition, we have shown that the regulation of DRG1 levels by direct DFRP1 binding is evolutionary conserved from sponges to humans, since the human DFRP1 recognizes the sponge DRG1, and vice versa. When co-transfected in different tumor cell lines, co-localization of DRG1 and DFRP1 was previously shown in the cytosol of human tumor cells HeLa S3, mouse 3T3 cells and *Drosophila melanogaster* cells^5,55,56^. On the contrary, some other studies show that DRG1 is localized in the nucleus of H1299 and A549 or HeLa cells^13^, or even in both of these compartments in HeLa cells^57^. Although the intracellular localization of DRG1 can be cell specific and/or cell-cycle phase dependent, there are contradictory findings even in frequently used HeLa cells. Therefore, we wanted to clarify the subcellular localization of DRG1. Our results confirm the localization of the sponge and the human DRG1 and DFRP1 in the cytosol of both MCF-7 and HeLa cells, similar to the results of several other studies^5,55,56^. To our knowledge, this is the first study that has shown a high conservation of sponge and human DRG1 biological features, especially regarding localization and necessity of DFRP1 binding, indicating their important function in analogous cellular pathways. Studies we conducted on α-tubulin dynamics show that DRG1 is also implicated in regulation of microtubules, polymers of α- and β-tubulin. Microtubules are an integral part of the cytoskeleton with a key role in cell division as they are responsible for chromosome segregation. Overexpression of DRG1 increases the number of multinucleated cells and chromosomal lagging, causing abnormal chromosome segregation due to deregulated microtubules and the spindle assembly checkpoint^13^. In addition, DRG1 and DFRP1 interact with microtubules in vitro, which can promote their polymerization, drive microtubule formation into bundles and stabilize microtubules. Interestingly, the proposed functions of DRG1 did not require its GTPase activity. Although spindles still formed during the mitosis when DRG1 was depleted, there was a delay in transition from prophase to anaphase in HeLa cells, and from aster to a spindle formation. This suggests that DRG1 might be required for optimal spindle dynamics during cell division^11^. We have shown that both the sponge and the human DRG1 and DFRP1 colocalize with endogenous α-tubulin suggesting the binding of DRG1 and α-tubulin in vivo. These results, together with the findings that the co-overexpression of the sponge or human DRG1 with the sponge or the human DFRP1 causes downregulation of α-tubulin, indicate that the human tumor cells might respond to higher levels of DRG1 by lowering α-tubulin expression. This, once again, highlights the interconnection between DRG1 and α-tubulin. Although our study confirms the role of DRG1 in proper microtubules localization and function, the mechanisms of DRG1 binding and its regulation of microtubules are unknown. Moreover, by intra- and interspecies co-expression of DRG1 and DFRP1, we have shown that the function of DRG1 in α-tubulin dynamics is evolutionary conserved from sponges to humans, indicating the importance of this regulation. DRG1 has been implicated in various tumors, including melanoma, osteosarcoma and lung cancer, as well as in various tumor cell lines. Studies have shown that DRG1 has a role in cell growth, proliferation, cell migration and colony formation^12,13,46^. In this study, we have shown that overexpression of sponge and human DRG1 increases cell proliferation, which is in accordance with other studies. For example, DRG1 knockdown in two osteosarcoma cell lines caused reduced cell viability and colony formation as well as an increase in apoptosis and G2/M arrest^46^. DRG1 is also overexpressed in melanoma, where its knock-down reduces cell proliferation and soft agar colony formation^12^. Additionally, overexpressed sponge and human DRG1 increases cell migration, which is consistent with the findings that DRG1 deficiency lowers cell migration and colony-formation in osteosarcoma cells^46^. Taken together, it is clear that the characteristics and biological function of DRG1 have been conserved throughout the metazoan evolution, from sponges to humans. The fact that sponges do not have cancer emphasizes the importance of DRG1 in fundamental cellular pathway(s).

## Conclusions

Our study demonstrates that (i) DRG1 gene/protein is highly conserved from sponge to humans. (ii) The sponge DRG1 has identical properties as human DRG1. Both recombinant sponge and human DRG1 are present in predominantly monomeric form and they form complexes with binding partner DFRP1. The ratio of DRG1:DFRP1 in the complex is 1:3 or 1:4. (iii) Sponge DRG1 possesses the intrinsic GTPase activity, which is enhanced by DFRP1. (iv) Sponge and human DRG1 bind nonspecifically to RNA and DNA. (v) Biological features, especially localization, the necessity of DFRP1 binding, and function of DRG1 in α-tubulin dynamics, are highly conserved between the sponge and human DRG1. (vi) The sponge DRG1 protein enhances proliferation, increases migration and colonization in human MDA-MB-231 cells, same as its human homolog. This study shows that the ancestor of all Metazoa already possessed a functional DRG1 gene/protein homolog, and that many of its multiple functions existed before the appearance of true tissues and origin of tumors. Biological functions of DRG1 and their biochemical background were established early in the metazoan evolution or even earlier in the evolution of life. Our results indicate that the ancestor of all animals possessed DRG1 protein with the structure and function similar to its evolutionarily recent versions present in the most complex extant animals.

## Methods

### Sequence analyses

The homologs of human DRG1 and DRG2 were identified in a variety of organisms at the National Center for Biotechnology Information database (NCBI) using the blastp algorithm (https://blast.ncbi.nlm.nih.gov/Blast.cgi). Genomes were additionally searched in the Ensembl database (https://metazoa.ensembl.org/index.html) and the TAIR database (https://www.arabidopsis.org/index.jsp) for *Mnemiopsis leidyi* and *Arabidopsis thaliana drg* genes, respectively. Protein sequences from selected organisms (listed in Supplementary Table S2) were subjected to a multiple sequence alignment analysis conducted by the MUSCLE algorithm^58^. To resolve the phylogenetic relationships of the DRG subfamily of proteins among archaea and eukaryotes, a maximum-likelihood tree was estimated in the MEGA7 software^59^. Unlike the eukaryotes, archaea contain only one DRG that shows a similar identity with both human DRG proteins, hence it was used as an outgroup to root a tree. Maximum-likelihood tree was based on LG + G + I evolutionary model^60^, according to the results obtained by ProtTest^61^. To evaluate the robustness of the phylogenetic tree, a bootstrap analysis from 1000 replications was performed. Obtained multiple sequence alignment was used to generate an amino acid identity and similarity matrices via the Matrix Global Alignment Tool (MatGAT2.01 with BLOSUM62 scores^62^), presented in Supplementary Table S3. The summarized identity/similarity datasets of DRG1 proteins were visualized using a heat map conducted by Morpheus (https://software.broadinstitute.org/morpheus/). For the intron-mapping of *drg1* genes from selected metazoan species and choanoflagellate, nucleotide sequences (with indicated intron positions) were taken from the NCBI's genomic database (https://www.ncbi.nlm.nih.gov/genome/). The exact position and the phase of each intron was verified by manually.

### Molecular docking and structure analysis

The AutoDock Vina software was utilized in the docking calculation, and as a docking target we used the structure of EsuDRG1 predicted with the Alpha fold^38^. The docking target was previously protonated using the MolProbity^63^. The GTP structure was downloaded from the ZINC database^64^ and prepared using Ligprep from the Schrödinger program package (Schrödinger Release 2021-4**:** LigPrep, Schrödinger, LLC, New York, NY, 2021). The active site of the target DRG1 protein was treated as rigid except residues: S72, S76, K75, T77, N246, K247, D249, H271, Q250, which presumably interact with the GTP molecule. The grid size was set at 30 × 30 × 30 XYZ points centered at (-13.877, 16.691, -9.281) Å and the grid spacing was set to 1 Å. The docking was carried out with Autodock Vina^39^. The target and ligand final preparation, visualization of the grid box, and the docking results were done by utilizing the AutoDock tools^65^. The generated protein:ligand complex was geometrically optimised in Maestro from Schrödinger program package (Schrödinger Release 2021-4: Maestro, Schrödinger, LLC, New York, NY, 2021). Structure visualization and analysis were performed using UCSF Chimera^66^.

### Permits

Sponge sampling was performed with the permit from the Ministry of Economy and Sustainable Development, Croatia.

### RNA isolation and cDNA library preparation

The tissue from the sponge *Eunapius subterraneus* (Tounjčica cave, Croatia) was homogenized and the cells were isolated using Falcon 200, 100 and 40 µm Cell Strainers. Total RNA was subsequently isolated using the RNeasy Kit (QIAGEN) according to the manufacturer’s instructions. The cDNA library was prepared from total RNA isolated from *E. subterraneus* using the High Capacity cDNA Reverse Transcription Kit (Applied Biosystems™) according to the manufacturer’s protocols.

### Plasmid construction

Unpublished transcriptome of *E. subterraneus* for the homologs of human DRG1, DRG2 and DFRP1 was searched. Based on the identified sponge sequence, primers for the sponge DRG1 (EsuDRG1, OL692370) and DFRP1 (EsuDFRP1, OL692371) from the cDNA library (Supplementary Table S1) were designed. EsuDRG1 and EsuDFRP1 were amplified, sequenced and cloned into pET28b, pEGFP-N1, pmCherry-C1, and pcDNA3.1 vectors. The cDNA sequences of human DRG1 (HsaDRG1) and DFRP1 (HsaDFRP1) from commercially available products were cloned into the same vectors. The primers and restriction enzymes used for cloning of EsuDRG1, HsaDRG1, EsuDFRP1 and HsaDFRP1 are listed in Supplementary Table S1. The resulting constructs are His-, GFP-, CHERRY-, FLAG-, MYC-tagged depending on the experiment.

### Protein expression and purification

Recombinant sponge and human proteins were produced in the *E.coli* strain BL21 CodonPlus (DE3). Cells transformed with sponge or human pET28b-DRG1-His and pET28b-DFRP1-His were grown at 37 °C in TB/Kan medium to OD_600_ of 0.6, induced with 0.1 mM IPTG, and grown at 16 °C for 18 h. Afterwards, the cells were washed and incubated on ice for 30 min in lysis buffer (50 mM HEPES, pH 7.4, 400 mM NaCl, 5 mM MgCl_2_, 10% glycerol (v/v), 10 mM imidazole, 1 mg/mL lysozyme (Sigma-Aldrich), protease inhibitor cocktail (Roche Applied Science) and 25000 units/mL Benzonase® Nuclease (Sigma-Aldrich)) and sonicated for 8 × 30 s at 4 °C. The lysate was purified by centrifugation for 40 min at 13280×*g* and 4 °C and filtered through a 0.22 μm sterile membrane filter. The filtered solution was loaded onto a cobalt affinity resin column (Takara). His tagged proteins were eluted with 150 mM imidazole and concentrated in storage buffer (25 mM HEPES pH 7.4, 300 mM NaCl, 5 mM DTT, and 10% glycerol) using Amicon 10 kDa cutoff filters (Merck). Produced proteins were analysed by SDS–polyacrylamide gel electrophoresis.

### Size-exclusion chromatography

Size-exclusion chromatography was performed at Biocentar d.o.o., Zagreb, Croatia. Recombinant proteins (EsuDRG1-His, EsuDFRP1-His and HsaDRG1-His) were loaded onto size-exclusion Superdex 200 Increase 10/300 GL column (GE Healthcare), pre-equilibrated with 10 mM phosphate buffer, 140 mM NaCl, pH 7.4. Proteins were eluted at 0.5 mL/2 min or 0.5 mL/1 min using an Äkta avant 25 system (GE Healthcare) at 4 °C. Injection volume was 500 µL. The column was calibrated with Bio-Rad Gel filtration standards: Thyroglobulin (670 kDa), γ-globulin (158 kDa), Ovalbumin (44 kDa), Myoglobin (17 kDa), and Vitamin B21 (1.35 kDa).

### Intrinsic GTPase assay

The intrinsic GTPase activity of EsuDRG1 and HsaDRG1 recombinant proteins was measured using the GTPase-Glo Assay (Promega) according to the manufacturer’s guidelines. For optimization, the purified proteins were serially diluted in a GTPase/GAP buffer containing 2 µM GTP, and the assay was carried out for 120 min at 37 °C. Luminescence was measured using white flat bottom 384-well microplates (Greiner) on the Infinite M200 plate reader (Tecan). To investigate the effect of DFRP1 on DRG1 GTPase activity, an equimolar mixture of 1.2 μM EsuDRG1 and EsuDFRP1 was assayed as described and relative luminescence was measured.

### DNA-binding assay

The DNA binding ability of EsuDRG1 and HsaDRG1 was assayed in vitro as described^30,67,68^. Briefly, the reactions contained 200 ng of single-stranded circular DNA of bacteriophage ΦX174 (NEB, #N3023S) or double-stranded covalently closed circular form of ΦX174 (NEB, # N3021S). The amount of purified proteins in the reaction ranged from 100 to 1600 ng. The reactions were performed in 20 μL, containing 40 mM Tris–acetate (pH 7.5) and 1 mM EDTA incubated for 30 min at 37 °C. The products were analysed by gel electrophoresis at 3 V/cm in 0.5% agarose for 3 h in 40 mM Tris–acetate (pH 7.5), 1 mM EDTA running buffer.

### RNA-binding assay

The nonspecific RNA binding ability of DRG1 proteins was assayed in vitro as described^7,69^. Briefly, 0.5 mg of proteins were incubated in 100 µL of cold reaction buffer (10 mM HEPES–NaOH (pH 7.4), 100 mM NaCl, 2 mM MgCl_2_, 0.1% Triton X-100, 3 mM DTT) with a free poly(U) (Sigma-Aldrich) at concentrations of 0, 0.1 and 1 mg/mL. The reactions were incubated for 30 min at 4 °C in the rotator. Subsequently, 10 µL of 50% poly(U)-agarose (Sigma Aldrich) in binding buffer was added to each reaction mixture and incubated for 30 min at 4 °C. The beads were washed six times in binding buffer following centrifugation for 1 min at 4 °C. Finally, proteins bound to the poly(U)-agarose were eluted by adding 10 µL of 4 × SDS sample buffer and boiled at 95 °C for 6 min. Samples were loaded onto a 12% SDS-PAGE gel and visualized by Coomassie brilliant blue.

### Protein cross-linking with glutaraldehyde

Protein cross-linking with glutaraldehyde was performed as described^30,69^ with the following modifications. The reactions containing 1 μg of DRG1 proteins alone or a 2 μg mixture of each of the EsuDRG1 and EsuDFRP1 protein were preincubated in PBS for 15 min. DRG1 proteins were crosslinked with 0.063%, 0.0125% and 0.025% glutaraldehyde for 5 min, and DRG1 and DFRP1 mixture was incubated with 0.125%, 0.25% and 0.5% glutaraldehyde for 30 min. The reactions were quenched with 0.2 M Tris–HCl, pH 7.5 for 15 min. All steps were performed at room temperature. The reaction products and untreated proteins (control) were boiled at 95 °C for 5 min, loaded onto 12% SDS-PAGE gel and visualized by staining with Coomassie brilliant blue. The same was repeated with human homologs.

### Cell culture and transient transfection

Human breast/mammary cancer cells MCF-7 (ECACC cat. no. 86012803) and MDA-MB-231 (ATCC cat. no. HTB-26) and cervical cancer cells HeLa (ATCC cat. no. CCL-2) were maintained in the Dulbecco’s Modified Eagle Medium with high glucose (DMEM, Sigma-Aldrich) supplemented with 10% fetal bovine serum (FBS, Capricorn Scientific), 1% nonessential amino acids (Sigma-Aldrich) and 1% antibiotic/antimycotic solution (Capricorn Scientific) in the humidified chamber at 37 °C with 5% CO_2_. Twenty-four hours after seeding, cells were transfected using Lipofectamine 3000 (Thermo Fisher Scientific) according to the manufacturer’s protocol and incubated for additional 24 h.

### Cell lysates preparation and Western blot

For protein level analysis, 5 × 10^5^ of MCF-7 cells were seeded in a six-well plate, transfected with expression vector pcDNA3 for FLAG-tagged sponge (EsuDRG1-FLAG) or human DRG1 (HsaDRG1-FLAG) and/or MYC-tagged sponge (EsuDFRP1-MYC) or human DFRP1 (HsaDFRP1-MYC), respectively. For Western blot analysis, cell lysates were prepared as follows: the cells were washed three times in PBS (pH 7.5) and homogenized in RIPA buffer (50 mM Tris pH 8.0, 150 mM NaCl, 5 mM EDTA, 1% NP40, 0.1% SDS, 0.5% sodium deoxycholate) containing a protein inhibitor cocktail (Roche Applied Science) and centrifuged at 16 000 × *g* for 10 min at 4 °C. The total protein concentration was measured using the commercially available Pierce BCA Protein Assay Kit (Thermo Fisher Scientific) according to the manufacturer’s protocol. Cell lysates were mixed with 6 × sample buffer (60% glycerol, 12% SDS, 3% DTT, 1/8 v/v 0.5 M Tris pH 6.8, bromophenol blue) and heated at 70 °C for 10 min. Equivalent amounts of protein were loaded onto Tris–Glycine gels. After SDS-PAGE, proteins were electrotransferred onto a PVDF membrane (Roche Applied Science) and incubated in primary antibody solution. Exogenous proteins were detected with anti-FLAG (clone M2, Sigma-Aldrich) and anti-MYC (clone 9E10, Sigma-Aldrich) antibodies, and endogenous proteins were detected with polyclonal antibodies against DFRP1 (Thermo Fisher Scientific) and DRG1 (Abcam) and monoclonal α-tubulin antibody (clone DM1A, Sigma-Aldrich). After incubation with primary antibodies, the membranes were washed and incubated in the HRP-conjugated secondary antibody (Bio-Rad) solution. Proteins were visualized by chemiluminescence using ECL blotting substrate (GE Healthcare) on a documentation system from UVItec Cambridge. AmidoBlack (Sigma-Aldrich) staining of membranes was used as a loading control, according to the manufacturer’s protocol. Protein signals were quantified using the ImageJ software (National Institutes of Health).

### Immunocytochemistry and confocal microscopy

For immunocytochemistry, MCF-7 (5 × 10^4^ cells/well) and HeLa cells (2 × 10^4^ cells/well) were seeded on sterile glass coverslips in a 24-well plate to achieve 70% confluence. After 24 h, cells were co-transfected with the fluorescently labelled sponge (EsuDRG1-GFP) or human DRG1 (HsaDRG1-GFP) together with sponge (EsuDFRP1-CHERRY) or human DFRP1 (HsaDFRP1*-*CHERRY). Control cells were co-transfected with empty-GFP (pEGFP-N1) and empty-CHERRY (pmCherry*-*C1) vector, respectively. Immunocytochemistry on MCF-7 cells was performed as previously described^70^. In short, the cells grown on coverslips were washed three times in PBS, fixed with 4% sucrose/paraformaldehyde for 15 min and permeabilized with 0.2% saponin in PBS. The cells were blocked in 4% donkey serum (Sigma-Aldrich) for 1 h at room temperature and subsequently incubated overnight with the primary antibody against α-tubulin (clone DM1A, Sigma-Aldrich) diluted in a blocking solution, followed by incubation in fluorescently labelled secondary antibody (Thermo Fisher Scientific) for 1 h. Hoechst (Sigma-Aldrich) was used to counterstain nuclei. Confocal images were acquired using the laser scanning confocal microscope Leica TCS SP8 (Leica Microsystems, Wetzlar, Germany). Additional image processing was performed by the ImageJ software (National Institutes of Health).

### Co-immunoprecipitation

For detection of DRG1 and DFRP1 complexes, 5 × 10^5^ of MCF-7 cells were seeded in a six-well plate, co-transfected with EsuDRG1-FLAG or HsaDRG1-FLAG and EsuDFRP1-MYC or HsaDFRP1-MYC. Briefly, cells were collected 24 h after transfection, lysed in coIP buffer (50 mM Tris pH 7.4, 150 mM NaCl, 2 mM EDTA, 1% NP40, 0.5% Triton X-100), supplemented with protease inhibitor cocktail (Roche Applied Science) and centrifuged at 16 000 × g for 10 min at 4 °C. Total protein concentration was measured using the commercially available Pierce BCA Protein Assay Kit (Thermo Fisher Scientific) according to the manufacturer’s protocol. Immunoprecipitation of FLAG-tagged EsuDRG1 or HsaDRG1 was performed using the anti-FLAG M2 affinity gel (Sigma-Aldrich) according to the manufacturer’s protocol. Briefly, 40 µL of gel was washed and incubated with 200 µg of proteins on a rotator at 4 °C overnight. The next day, the samples were centrifuged at 800 × *g* for 15 min at 4 °C, supernatant was saved, and the complexes were washed two times in a buffer containing 50 mM Tris pH 7.6, 500 mM NaCl, 2 mM EDTA, and two times in buffer with 50 mM Tris pH 7.6, 150 mM NaCl, 2 mM EDTA. Between each washing step, samples were centrifuged at 800 × g for 1 min at 4 °C. Complexes were resuspended in a sample buffer (20% glycerol, 4% SDS, 1% DTT, 1/24 v/v 0.5 M Tris pH 6.8, bromophenol blue), separated by SDS-PAGE and electrotransferred onto PVDF membrane (Roche Applied Science). DRG1 and DFRP1 complexes were detected using the primary antibodies against FLAG (clone M2, Sigma-Aldrich) and MYC (clone 9E10, Sigma-Aldrich), and visualized by chemiluminescence using ECL blotting substrate (GE Healthcare) on a documentation system from UVItec Cambridge.

### MTT assay

Cell proliferation was monitored using the MTT assay. Briefly, 7 × 10^3^ of MCF-7 cells and 4 × 10^3^ of MDA-MB-231 cells were seeded in a 96-well plate and transfected with EsuDRG1-FLAG and/or HsaDRG1-FLAG and/or EsuDFRP1-MYC or HsaDFRP1-MYC. Forty-eight h after the transfection growth medium was removed, 1 × MTT was added and cells were incubated for 4 h in the growth conditions, followed by an addition of dimethyl sulphoxide and 2 min incubation with gentle mixing. The absorbance was measured at 570 nm on ELISA microplate reader (LabSystem Multiskan MS, Artisan Technology Group).

### Colony formation assay

To test colony formation, MDA-MB-231 cells were co-transfected with EsuDRG1-FLAG or HsaDRG1-FLAG and EsuDFRP1-MYC or HsaDFRP1-MYC. Twenty-four h after the transfection cells were resuspended and seeded in 60 mm dishes at 5 × 10^4^ cells/dish and G418 (Neomycin, Sigma-Aldrich) was added to a final concentration of 500 µg/mL for the selection of resistant colonies. After 10 days, the resistant colonies were fixed with 100% methanol for 10 min, dried, stained with 10% Giemsa (Sigma-Aldrich) for 30 min and counted.

### Wound healing assay

The MDA-MB-231 (5 × 10^4^ cells/well) cells were seeded in a 24-well plate, co-transfected with EsuDRG1-FLAG or HsaDRG1-FLAG and EsuDFRP1-MYC or HsaDFRP1-MYC. Twenty-four h after transfection, a wound by scratching the cell monolayer in a straight line was made with a sterile 100 μL tip. The cells were washed with fresh medium and incubated for 24 h. Cell migration was monitored by measuring the distances between the two margins of the scratch after 24 h in five fields of each chamber using 100× magnification on the microscope (Olympus CKX41, Tokyo, Japan). Distances were quantified by comparing the distances at the time point zero and after 24 h in the same field using the ImageJ software (National Institutes of Health).

### Cell migration assay

For monitoring cell migration MDA-MB-231 cells were co-transfected with EsuDRG1-FLAG or HsaDRG1-FLAG and EsuDFRP1-MYC or HsaDFRP1-MYC. The cells were seeded in a migration Transwell cell culture inserts (pore size 8 mm; Corning) at a density of 2.5 × 10^4^ cells/well, and left to migrate for 24 h towards 10% FBS in DMEM as a chemoattractant. The cells that migrated to the underside of the filter were fixed with 4% PFA and stained with 1% crystal violet solution. Images were acquired at a 200× magnification using the microscope (Olympus BX51, Tokyo, Japan) and quantified by the ImageJ software (National Institutes of Health, USA).

### Statistical analysis

All biological experiments were repeated at least three times in biological duplicates or triplicates. Statistical analysis was performed using the SPSS statistical package for Windows, v17. For the determination of statistically significant differences between the means of three or more groups, one-way ANOVA with appropriate *post-hoc* analyses was used. To determine if a difference exists between the means of two independent groups, the *t-test* was performed. The statistical significance of the tests was set at *p* < 0.05.

### Consent for publication

All authors read and approved the final manuscript. All authors confirmed that submitted manuscript has neither been published, nor simultaneously submitted elsewhere.

## Supplementary Information

Supplementary Information 1.

Supplementary Information 2.

Supplementary Information 3.

## Data Availability

All the data generated or analysed during this study are included in this published article and its supplementary information files.
