# Supplementary material for: Structure and function of cancer-related developmentally regulated GTP-binding protein 1 (DRG1) is conserved between sponges and humans
Source: Sci Rep. 2022 Jul 5;12:11379. doi: 10.1038/s41598-022-15242-2 (PMC9256742; doi:10.1038/s41598-022-15242-2)
Supplement: Supplementary file 1 — Supplementary Information 1. [file 41598_2022_15242_MOESM1_ESM.pdf]

Structure and function of cancer-related Developmentally regulated GTP-binding protein 1 (DRG1) is conserved between sponges and humans

Authors: Silvestar Beljan[1,2,#], Kristina Dominko[1,#], Antea Talajić[1,#], Andrea Hloušek-Kasun[1], Nikolina Škrobot Vidaček[1], Maja Herak Bosnar[3], Kristian Vlahoviček[2], Helena Ćetković[1]*

Affiliations:

[1]Division of Molecular Biology, Ruđer Bošković Institute, 10000 Zagreb, Croatia

[2]Division of Biology, Faculty of Science, University of Zagreb, 10000 Zagreb, Croatia

[3]Division of Molecular Medicine, Ruđer Bošković Institute, 10000 Zagreb, Croatia

***Corresponding author:** Helena Ćetković, Division of Molecular Biology, Ruđer Bošković Institute, Bijenička cesta 54, 10000 Zagreb, Croatia

E-mail: cetkovic@irb.hr; Tel.: +385-1-4561115

[#]SB, KD and AT contributed equally to this work.

This file includes:

- **Figure S1-S9** (Supplementary figures)
- **Table S1** (List of primers and constructs used in the study)
- **Figure S10** (Original images of gels and western blots before cropping)

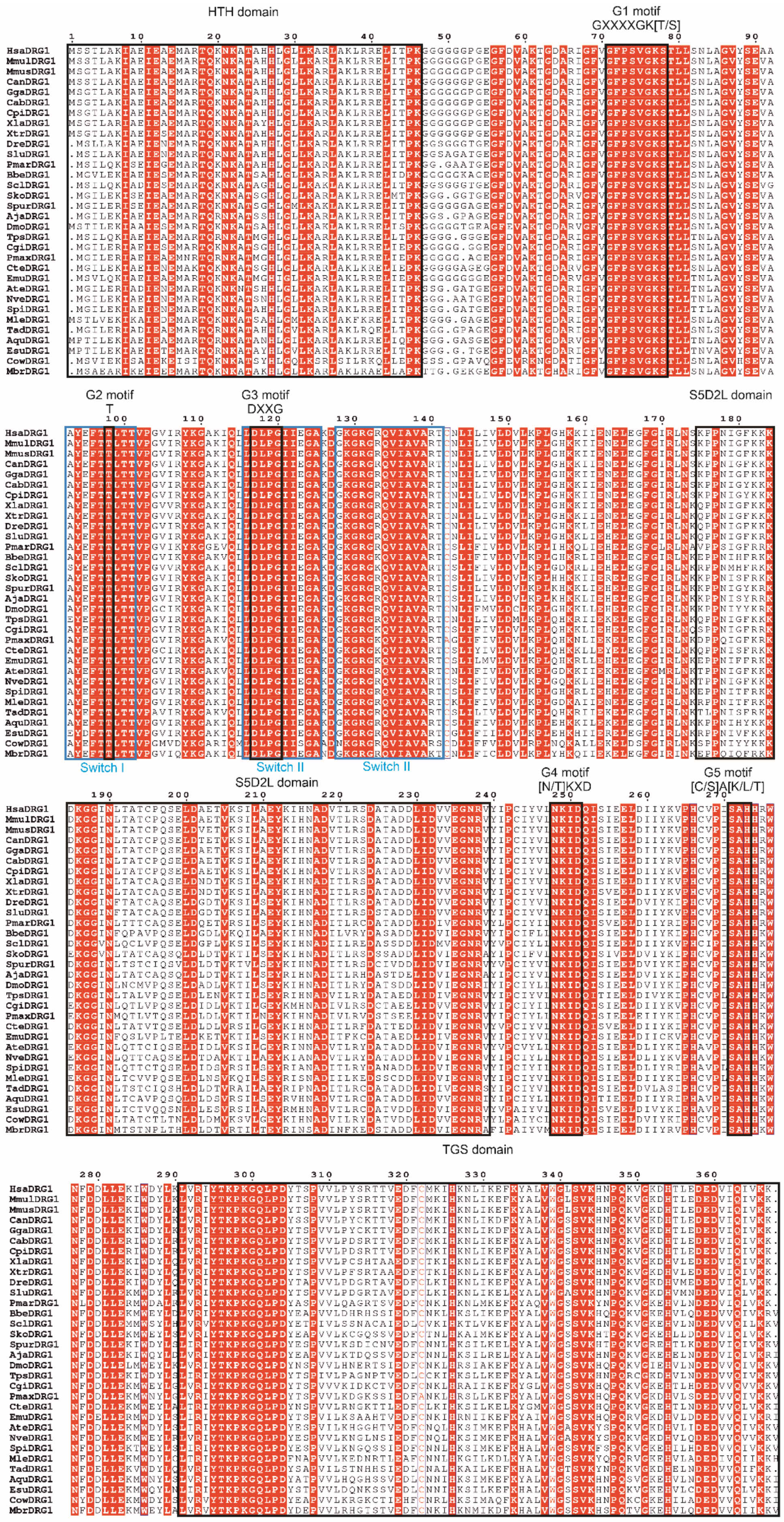
HTH domain
G1 motif
GXXXXGK[T/S]
G2 motif
T
G3 motif
DXXG
S5D2L domain
Switch I
Switch II
Switch II
S5D2L domain
G4 motif
[N/T]KXD
G5 motif
[C/S]A[K/L/T]
TGS domain

Fig. S1. Multiple sequence alignment of DRG1 homologs from representatives of Metazoa and their closest unicellular relatives. The conserved regions of DRG1 proteins are shown in boxes with the names of the domains indicated above the alignment, starting with the N-terminus; HTH domain, G1 motif, G2 motif, G3 motif, S5D2L domain, G4 motif, G5 motif and TGS domain with the corresponding motifs. The amino acid sequences of DRG1 were aligned using the ClustalX; the alignment was visualised using the ESPript. Red boxes with white letters indicate strict identity.

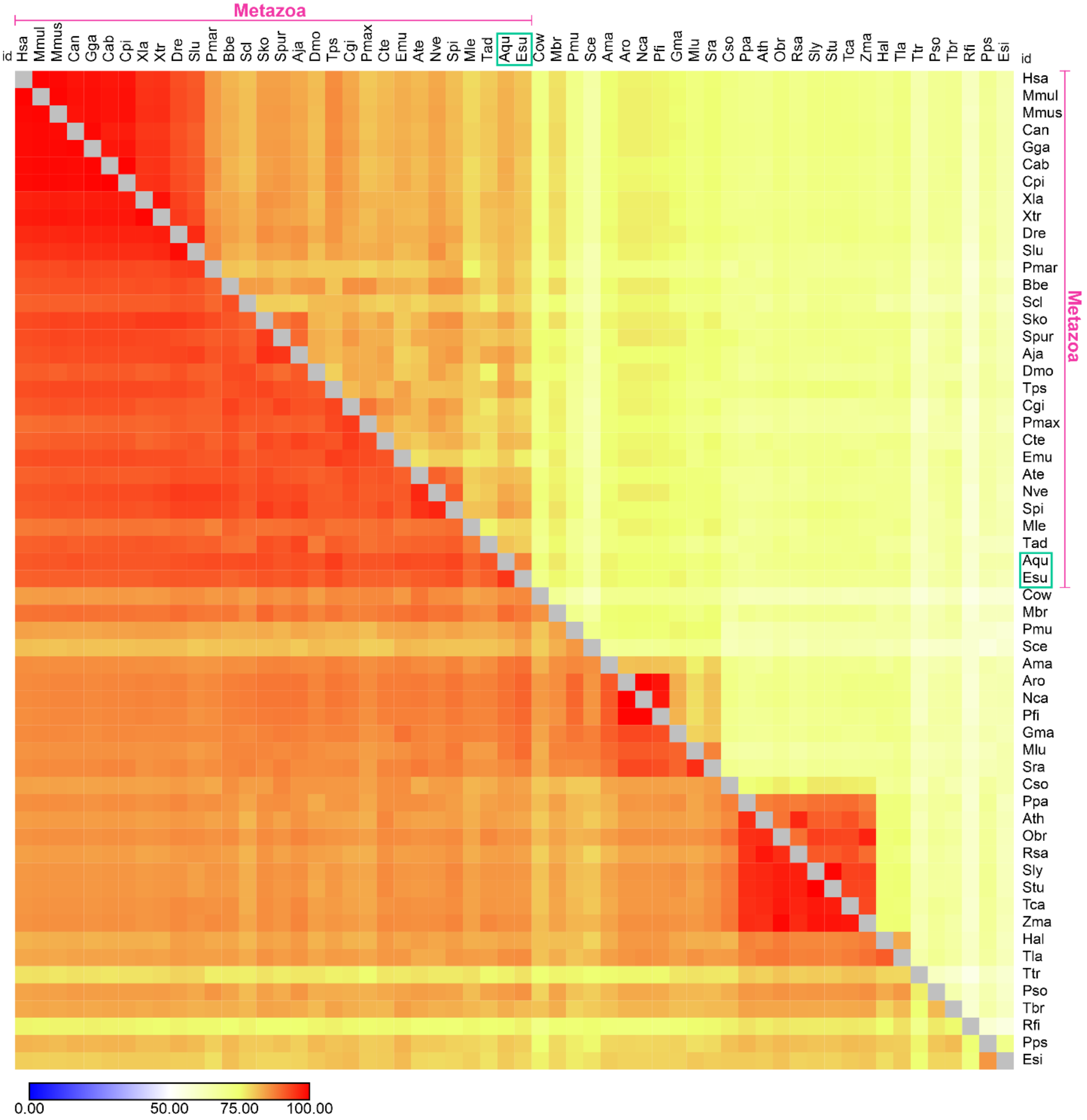

Fig. S2. Heatmap of amino acid sequence similarity (lower left) and identity (upper right) values for DRG1 proteins generated by Morpheus (https://software.broadinstitute.org/morpheus). Protein sequences accession numbers and supporting identity/similarity percentages matrices (MatGAT2.01 with Matrix BLOSUM62 scores) are presented in Supplementary Table S2 and S3. High amino acid similarity (>50%) is indicated by warm colors (yellow and red) and low similarity (<50%) in blue. Metazoan DRG1 proteins are indicated in pink. Sponges DRG1 proteins are indicated in green.

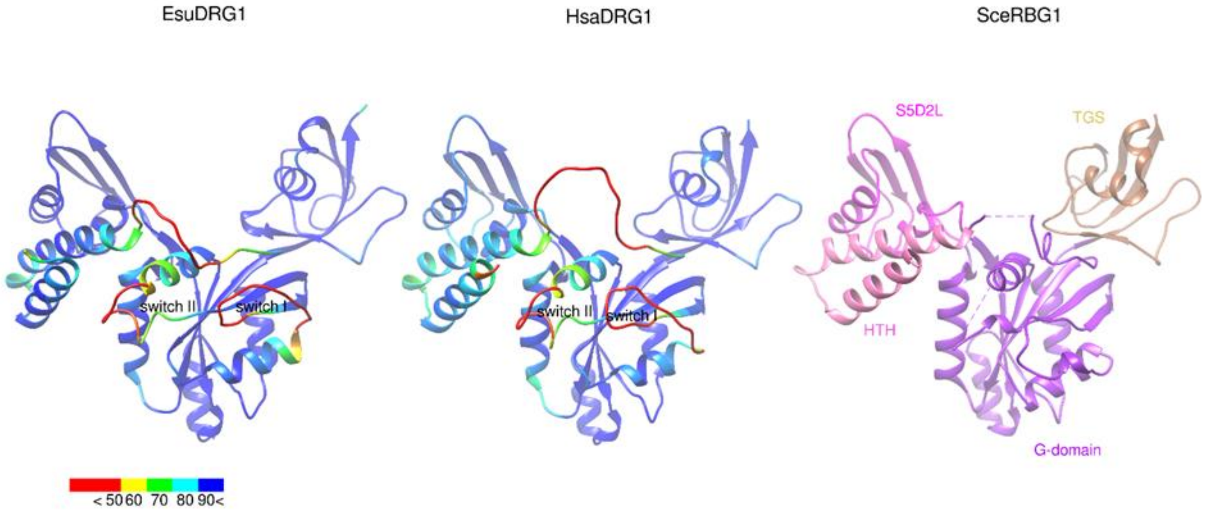

Fig. S3. Predicted 3D structures of DRG1 from *Eunapius subterraneus* and *Homo sapiens* compared to crystal structure of Rbg1 from *Saccharomyces cerevisiae*, SceRBG1 (PDB:4A9A). The secondary protein structures are colored by probability of the predicted atom position. Atoms that have the lowest probability of the predicted position are colored in red, while the highest probability is colored in dark blue.

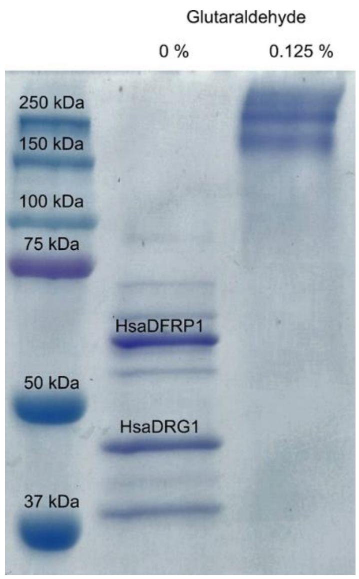

Fig. S4. Human DRG1 and DFRP1 form heterooligomers. Crosslinking of HsaDRG1+HsaDFRP1 with glutaraldehyde. Glutaraldehyde was added to a final amount of 0.125%. The reaction was incubated at 25 °C for 30 min.

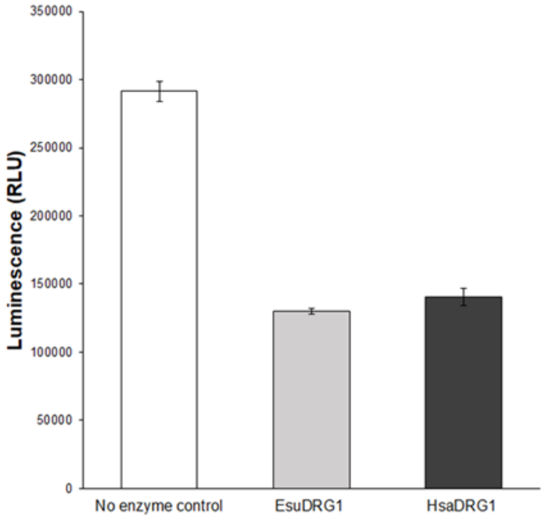

Fig. S5. Intrinsic GTPase activity of sponge and human DRG1 (concentration of 1.2 µM of each protein). The control sample contains only the GTP/GAP buffer. Standard deviations are indicated on the bars. RLU, relative luminescence unit.

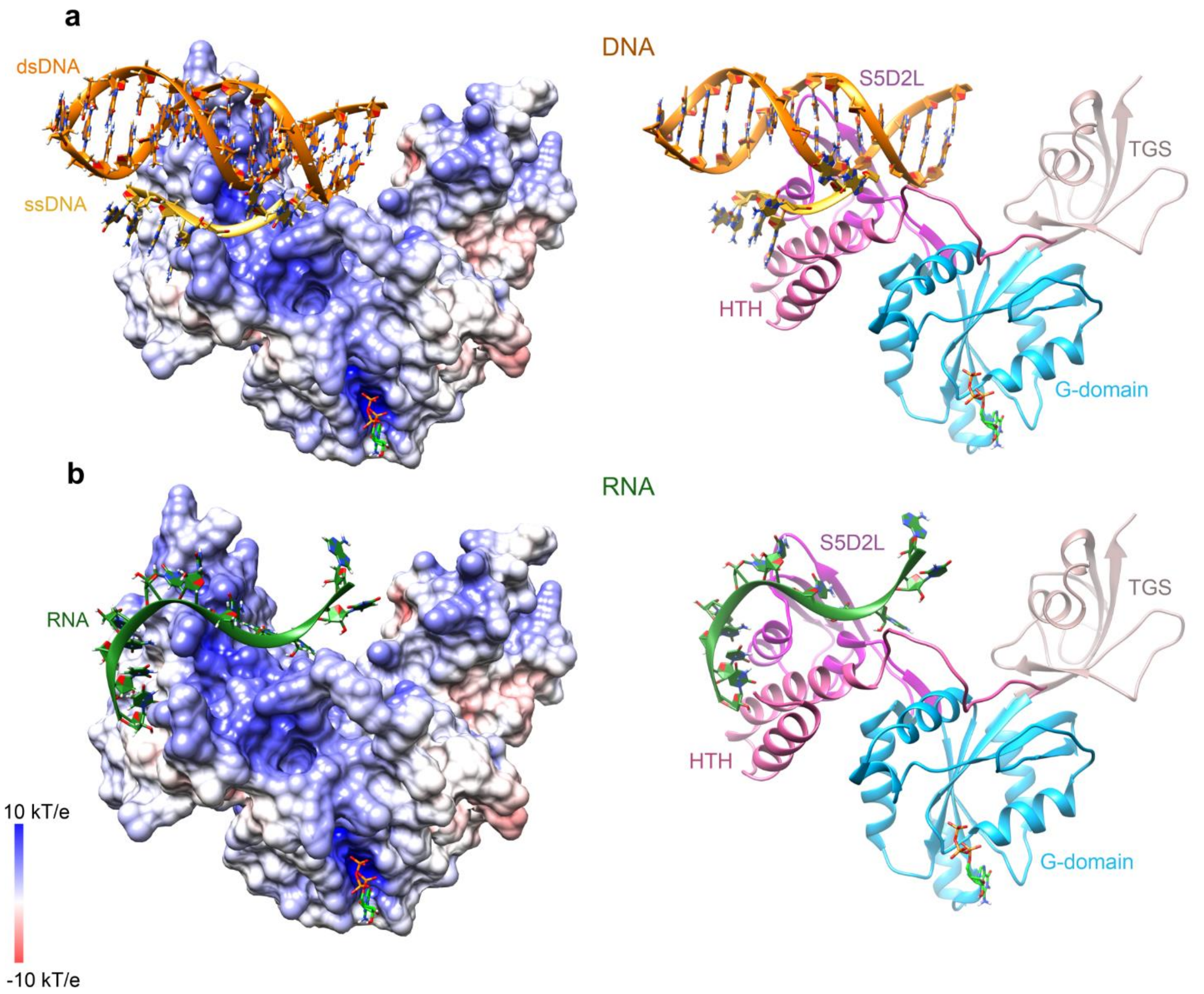

Fig. S6. EsuDRG1 is in complex with DNA, RNA and GTP.

EsuDRG1 in complex with a ds and ssDNA and b RNA. The electrostatic surface of DRG1 is calculated using APBS web server (https://server.poissonboltzmann.org/). The docking scores for the best-predicted poses are: 9.0 +/- 5.6 for RNA, 6.7 +/- 9.7 for ssDNA, and 32.5 +/-17.9 for dsDNA. Bound GTP is shown as sticks and colored green.

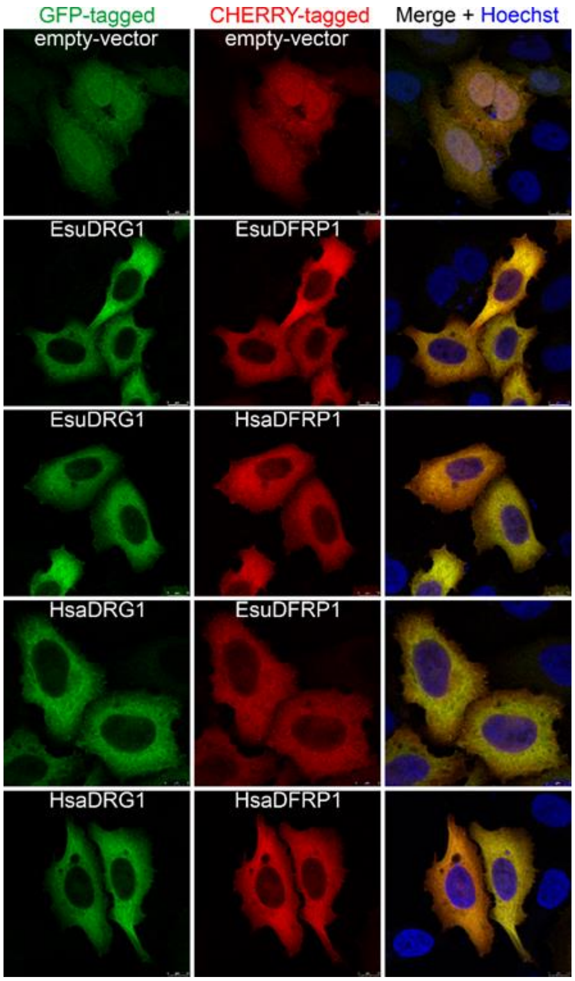

Fig. S7. Intracellular localization of both human DRG1 and its sponge homolog is in the cytosol of HeLa cells. Colocalization (yellow) of human DRG1 or its sponge homolog with human or sponge DFRP1 in the cytosol of HeLa cells. Human and sponge DRG1 were fluorescently labelled with GFP (green) and human and sponge DFRP1 with CHERRY (red). Hoechst was used to stain nuclei. The cells were analysed by confocal microscopy. The experiments were repeated three times in biological duplicates. Esu-sponge *Eunapius subterraneus*, Hsa-human.

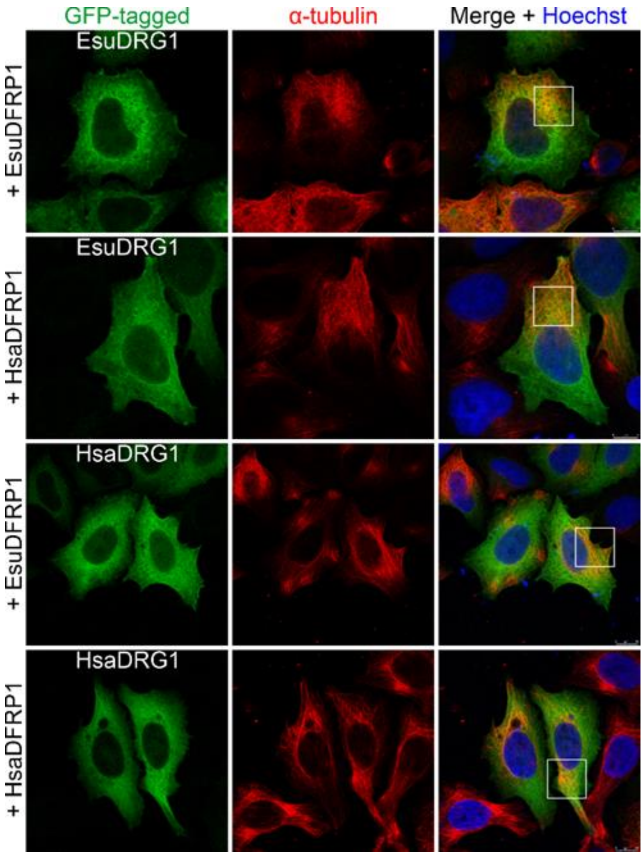

Fig. S8. Human and sponge DRG1 colocalized with α-tubulin in the cytosol of HeLa cells. Cells were transfected with human and sponge DRG1-GFP (green) and human and sponge DFRP1-CHERRY (not shown), fixed and stained with antibody against α-tubulin (red) and analysed by confocal microscopy. Colocalization of human and sponge DRG1-GFP (green) with α-tubulin (red) was depicted by yellow square. Hoechst was used to stain nuclei. The experiments were repeated three times in biological duplicates. Esu-sponge *Eunapius subterraneus*, Hsa-human.

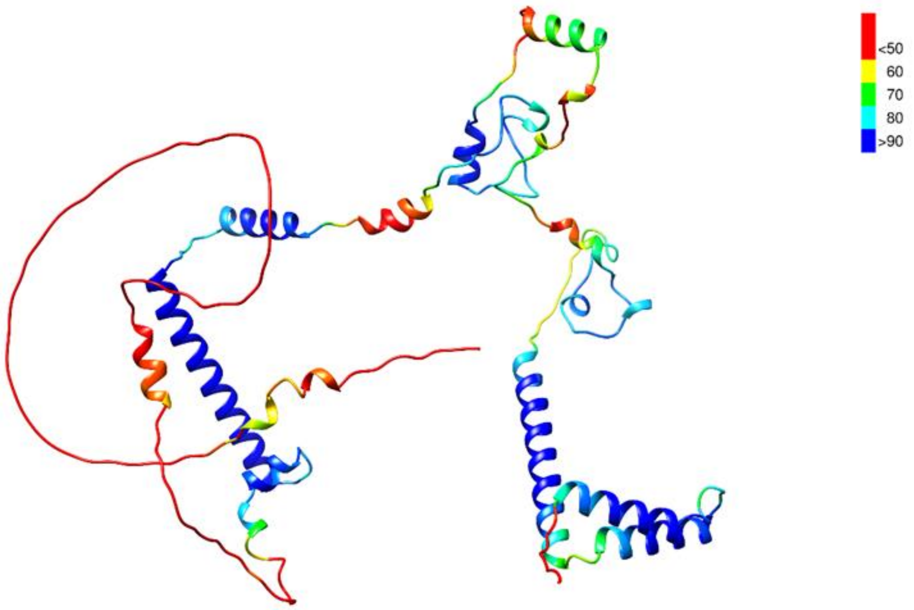

Fig. S9. Structures of DFRP1 from *Eunapius subterraneus predicted with Alpha fold.* The secondary structures are colored by probability of predicted atom positions. Atoms that have the lowest probability of predicted position are colored in red, while the highest probability is colored in dark blue.

Table S1. List of primers and constructs used in the study.

Construct Name/ Organism	Origin	Cloned in	Primers/restriction site	TAG	Host expression
**EsuDRG1**<br>E. subterraneus	Esu cDNA	/	5'- ATGCCTACGATACTTGAAAAGATAGCG -3'<br>5'- CAATTAGATTTTCTTAACGAGTTGAACGAC -3'	/	/
**EsuDFRP1**<br>E. subterraneus	Esu cDNA	/	5'- GAATGCCACCGAAGAAAGGTGCGC -3'<br>5'- CTCAATCTTCACCAGCCTCATCGTC -3'	/	/
**EsuDRG1-His**<br>E. subterraneus	EsuDRG1	pET28b	NdeI 5'- GTCTAG<u>CATATG</u>CCTACGATACTTGAAAAG -3'<br>BamHI 5'- CTAGAC<u>GGATCC</u>TTAGATTTTCTTAACGAGTTG -3'	6xHis<br>N ter	Bacterial
**EsuDFRP1-His**<br>E. subterraneus	EsuDFRP1	pET28b	NdeI 5'- GTCTAG<u>CATATG</u>CCACCGAAGAAAGGTGCGCAAGC -3'<br>BamHI 5'- CTAGAC<u>GGATCC</u>TCAATCTTCACCAGCCTCATCGTC -3'	6xHis<br>N ter	Bacterial
**EsuDRG1-GFP**<br>E. subterraneus	EsuDRG1-His	pEGFP-N1	XhoI 5'- GTCTAG<u>CTCGAG</u>ATGCCTACGATACTTGAAAAG -3'<br>BamHI 5'- CTAGAC<u>GGATCC</u>CGGGAGATTTTCTTAACGAGTTG -3'	GFP<br>C ter	Mammalian
**EsuDFRP1-CHERRY**<br>E. subterraneus	EsuDFRP1-His	pmCherry-C1	XhoI 5'- GTCTAG<u>CTCGAG</u>GCATGCCACCGAAGAAAGGTGCG -3'<br>BamHI 5'- CTAGAC<u>GGATCC</u>TCAATCTTCACCAGCCTCATCG -3'	Cherry<br>N ter	Mammalian
**EsuDRG1-FLAG**<br>E. subterraneus	EsuDRG1-His	pcDNA3.1	BamHI 5'- GTCTA<u>GGGATCC</u>ACGAGATGGACTACAAGGACGACGACGATAAGATGCCTACGATACTTG -3'<br>XhoI 5'- CTAGAC<u>CTCGAG</u>TTAGATTTTCTTAACGAG -3'	FLAG<br>N ter	Mammalian
**EsuDFRP1-MYC**<br>E. subterraneus	EsuDFRP1-His	pcDNA3.1	BamHI 5'- GTCTAG<u>GGATCC</u>ACGAGATGCCACCGAAGAAAGGTGCG -3'<br>XhoI 5'- CTAGAC<u>CTCGAGT</u>CACAGATCCTCTTCTGAGATGAGTTTCTGCTCGGAATCTTCACCAGCCTC -3'	Myc<br>C ter	Mammalian
**HsaDRG1-His**<br>H. sapiens	DRG1 pPB-N-His abm	pET28b	NdeI restriction enzyme<br>XhoI restriction enzyme	6xHis<br>N ter	Bacterial
**HsaDFRP1-His**<br>H. sapiens	HG23194-CM SinoBiological	pET28b	NdeI 5'- GTCTAG<u>CATATG</u>ATGCCCCCCAAGAAACAGGCTCAGGCCG -3'<br>EcoRI 5'- CTAGAC<u>GAATTC</u>TTATTCTTCTAAATCAAGTGTATTTAATTC -3'	6xHis<br>N ter	Bacterial
**HsaDRG1-GFP**<br>H. sapiens	HG14741-NF SinoBiological	pEGFP-N1	XhoI 5'- GTCTAG<u>CTCGAG</u>ATGAGCAGCACCTTAGCTAAG -3'<br>BamHI 5'- CTAGAC<u>GGATCC</u>CTGGACTTCTTCACAATTTG -3'	GFP<br>C ter	Mammalian
**HsaDRG1-CHERRY**<br>H. sapiens	HG14741-NF SinoBiological	pmCherry-C1	XhoI 5'- GTCTAG<u>CTCGAG</u>CTATGAGCAGCACCTTAGC -3'<br>BamHI 5'- CTAGAC<u>GGATCC</u>TCACTTCTTCACAATTTGAATGAC -3'	Cherry<br>N ter	Mammalian
**HsaDFRP1-CHERRY**<br>H. sapiens	HG23194-CM SinoBiological	pmCherry-C1	XhoI 5'- GTCTAG<u>CTCGAG</u>CCATGCCCCCCAAGAAACAGG -3'<br>BamHI 5'- CTAGAC<u>GGATCC</u>TTATTCTTCTAAATCAAGTG -3'	Cherry<br>N ter	Mammalian
**HsaDRG1-FLAG**<br>H. sapiens	HG14741-NF SinoBiological	pcDNA3.1	BamHI 5'- GTCTAG<u>GGATCC</u>ACGAGATGGACTACAAGGACGACGACGATAAGATGAGCAGCACCTTAGCTAAG -3'<br>XhoI 5'- CTAGAC<u>CTCGAG</u>TCACTTCTTCACAATTTG -3'	FLAG<br>N ter	Mammalian
**HsaDFRP1-MYC**<br>H. sapiens	HG23194-CM SinoBiological	pcDNA3.1	BamHI 5'- GTCTAG<u>GGATCC</u>ACGAGATGCCCCCCAAGAAACAGGC -3'<br>XhoI 5'- CTAGAC<u>CTCGAG</u>TCACAGATCCTCTTCTGAGATGAGTTTCTGCTCGGATTCTTCTAAATC -3'	Myc<br>C ter	Mammalian

a)

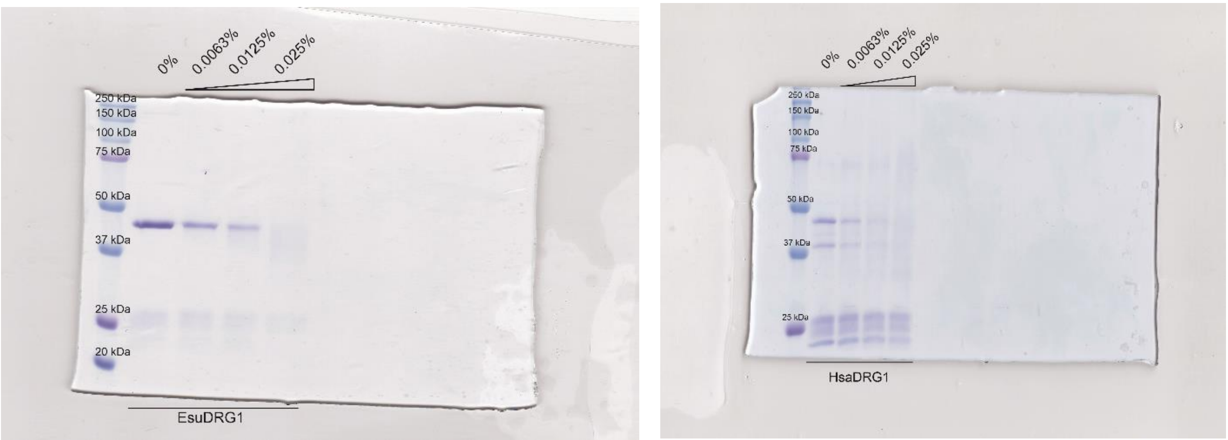
0%
0.0063%
0.0125%
0.025%
250 kDa
150 kDa
100 kDa
75 kDa
50 kDa
37 kDa
25 kDa
20 kDa
EsuDRG1
HsaDRG1

b)

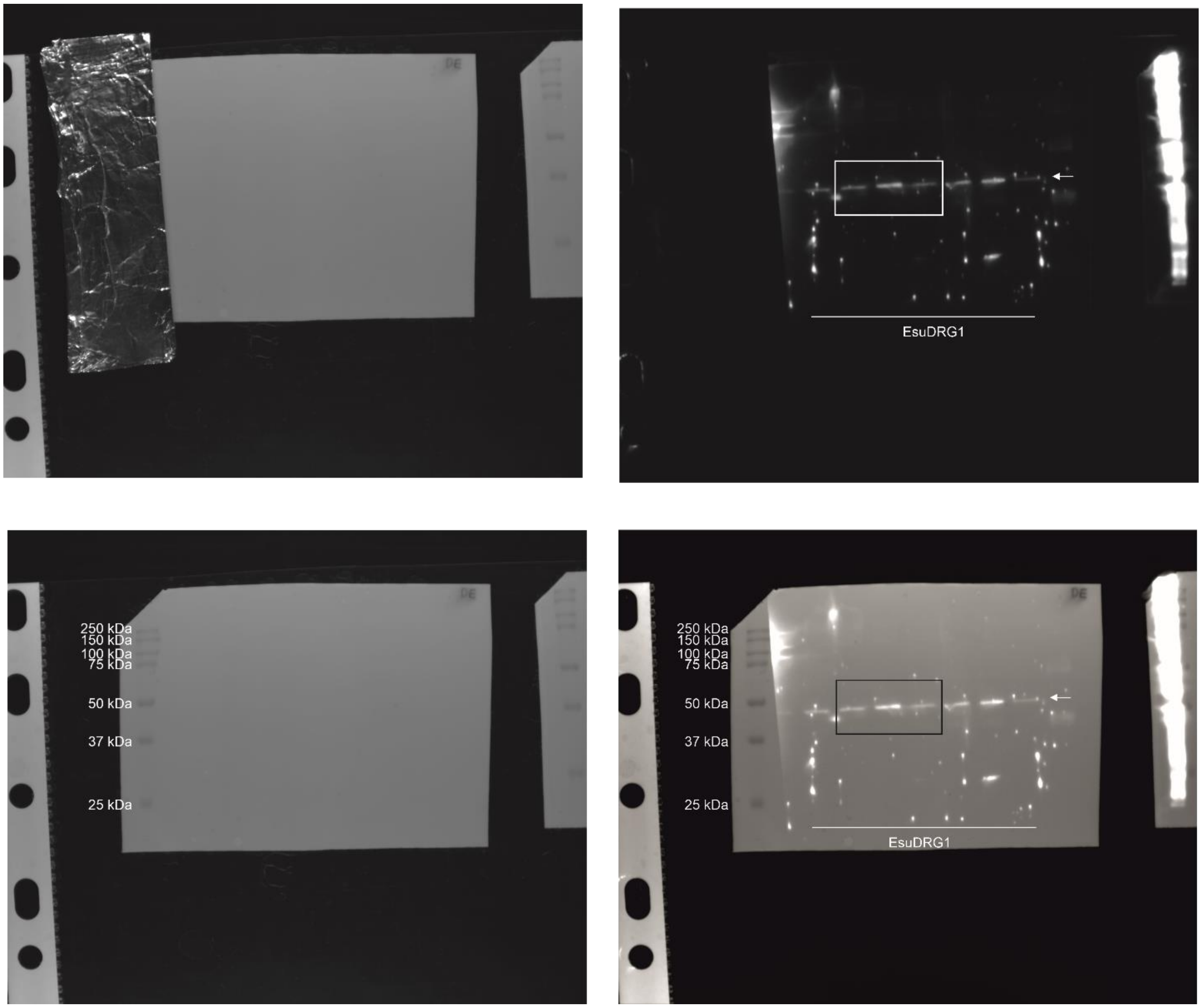
EsuDRG1
250 kDa
150 kDa
100 kDa
75 kDa
50 kDa
37 kDa
25 kDa
EsuDRG1

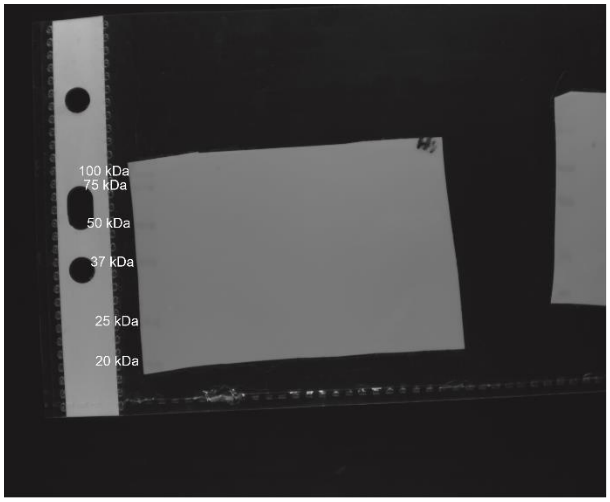
100 kDa
75 kDa
50 kDa
37 kDa
25 kDa
20 kDa

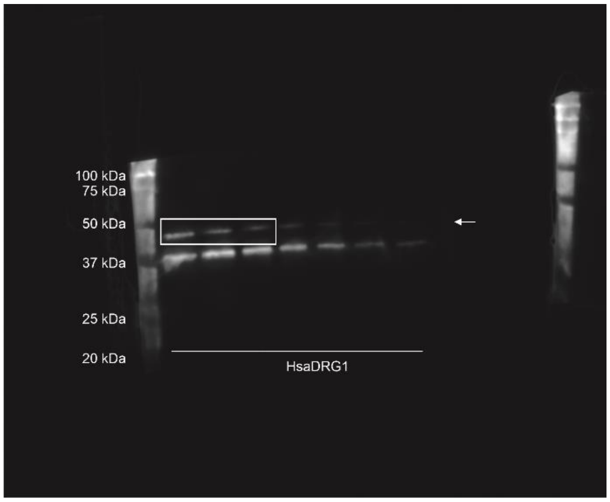
100 kDa
75 kDa
50 kDa
37 kDa
25 kDa
20 kDa
HsaDRG1

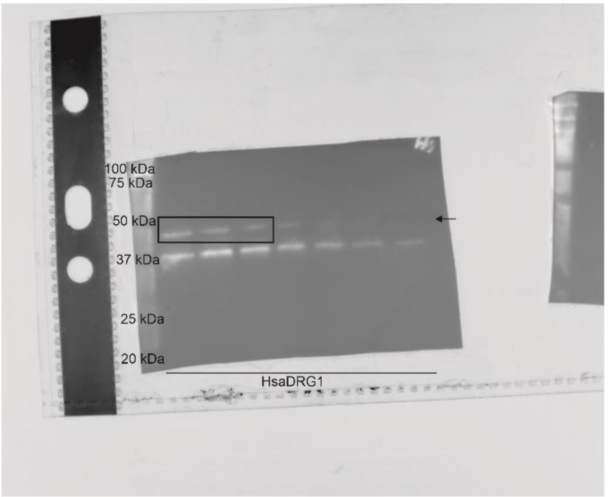
100 kDa
75 kDa
50 kDa
37 kDa
25 kDa
20 kDa
HsaDRG1

c)

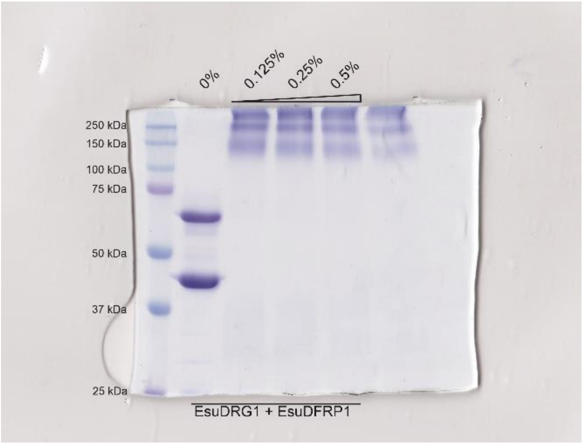
0%
0.125%
0.25%
0.5%
250 kDa
150 kDa
100 kDa
75 kDa
50 kDa
37 kDa
25 kDa
EsuDRG1 + EsuDFRP1

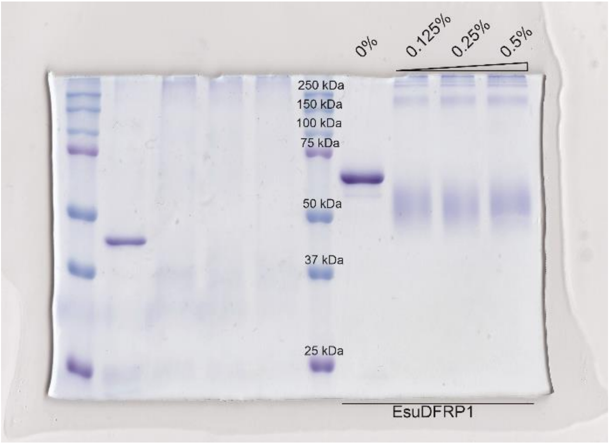
0%
0.125%
0.25%
0.5%
250 kDa
150 kDa
100 kDa
75 kDa
50 kDa
37 kDa
25 kDa
EsuDFRP1

d)

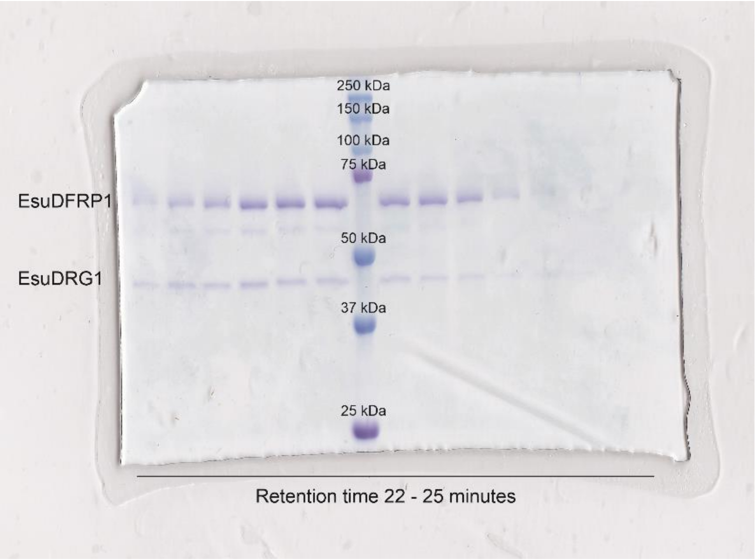
250 kDa
150 kDa
100 kDa
75 kDa
EsuDFRP1
50 kDa
EsuDRG1
37 kDa
25 kDa
Retention time 22 - 25 minutes

e)

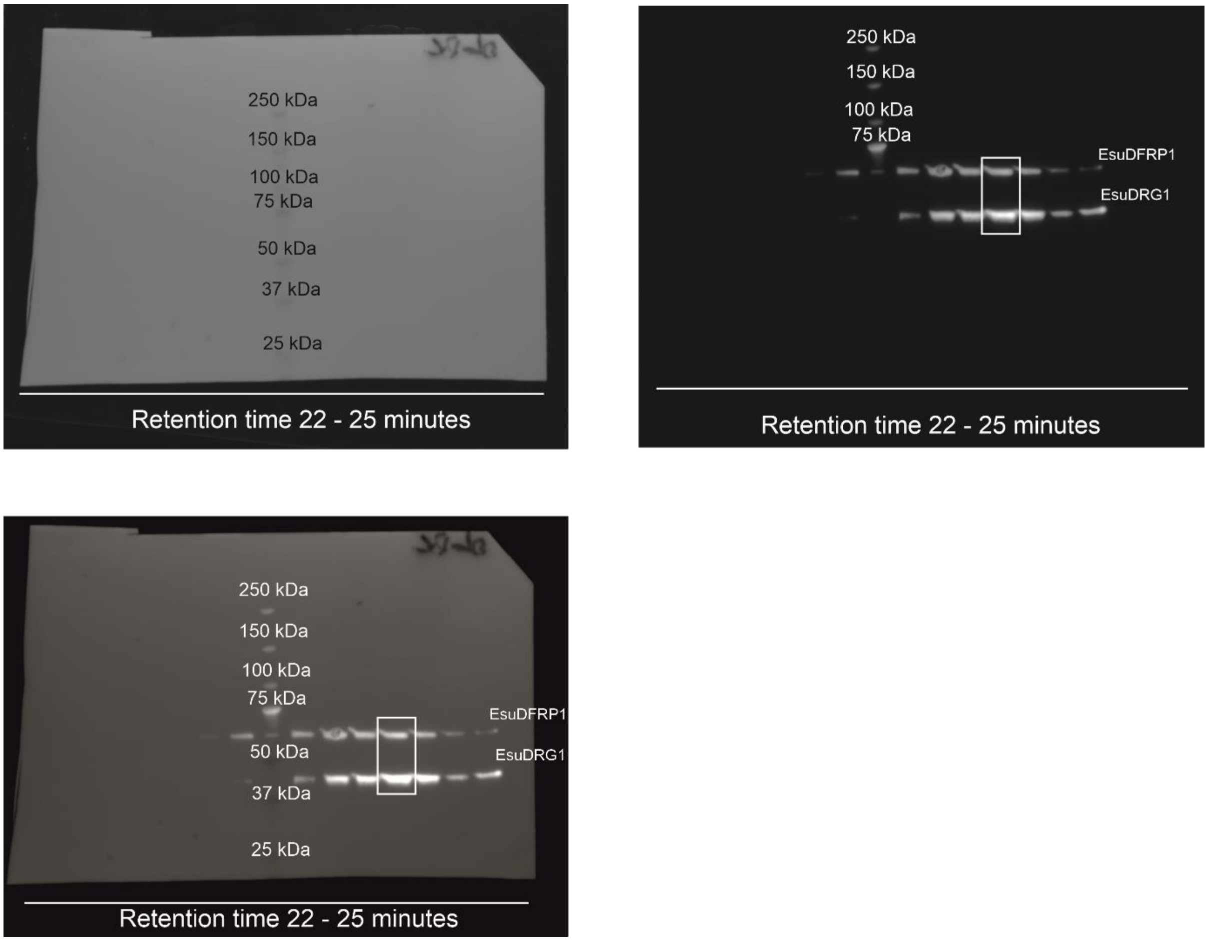
250 kDa
150 kDa
100 kDa
75 kDa
50 kDa
37 kDa
25 kDa
Retention time 22 - 25 minutes
250 kDa
150 kDa
100 kDa
75 kDa
EsuDFRP1
EsuDRG1
Retention time 22 - 25 minutes
250 kDa
150 kDa
100 kDa
75 kDa
EsuDFRP1
50 kDa
EsuDRG1
37 kDa
25 kDa
Retention time 22 - 25 minutes

f)

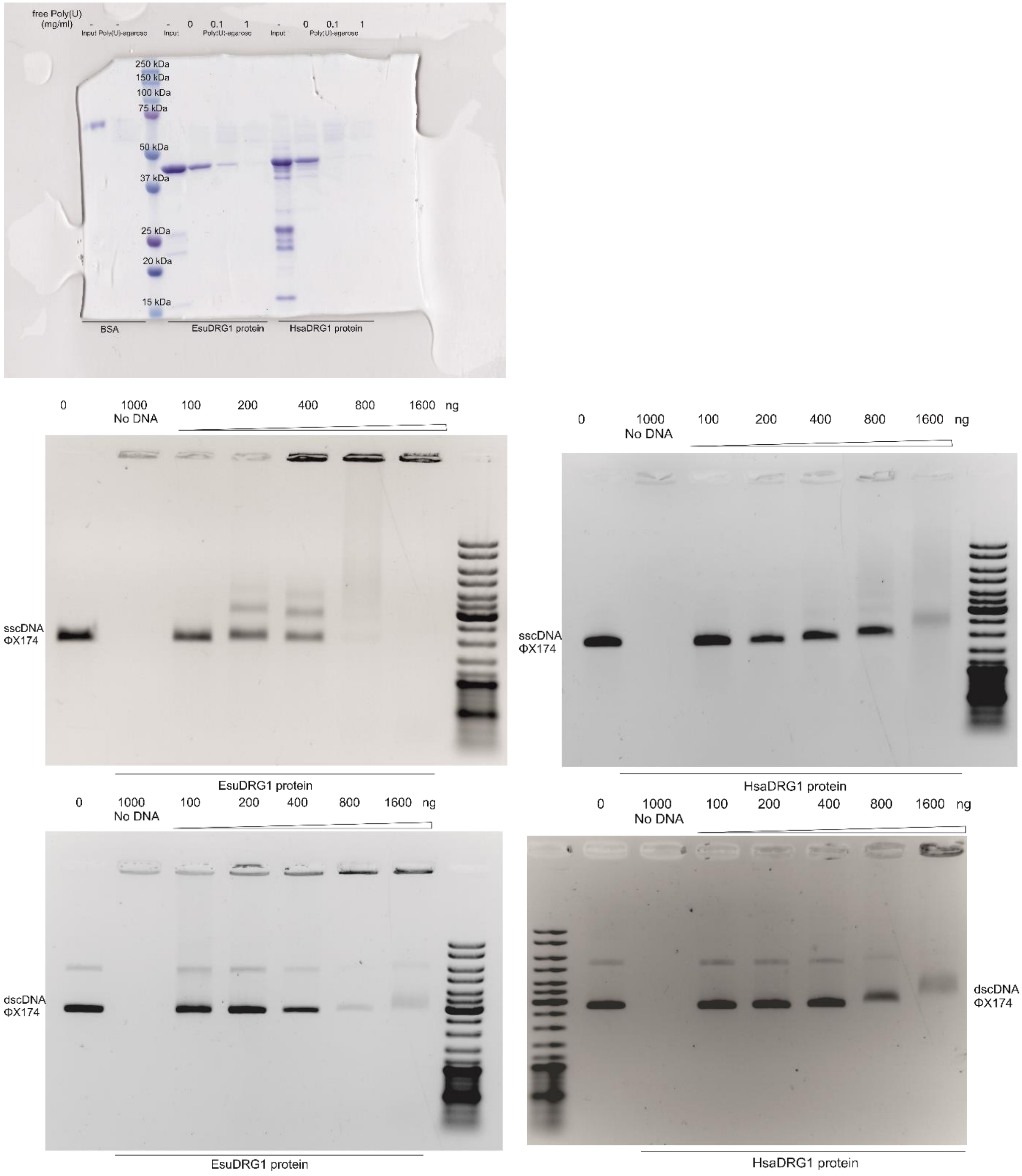
free Poly(U)
(mg/ml) - - - 0 0.1 1 - 0 0.1 1
Input Poly(U)-agarose
Input
Poly(U)-agarose
Input
Poly(U)-agarose
250 kDa
150 kDa
100 kDa
75 kDa
50 kDa
37 kDa
25 kDa
20 kDa
15 kDa
BSA
EsuDRG1 protein
HsaDRG1 protein
0 1000 No DNA 100 200 400 800 1600 ng
sscDNA ΦX174
EsuDRG1 protein
0 1000 No DNA 100 200 400 800 1600 ng
sscDNA ΦX174
HsaDRG1 protein
0 1000 No DNA 100 200 400 800 1600 ng
dscDNA ΦX174
EsuDRG1 protein
0 1000 No DNA 100 200 400 800 1600 ng
dscDNA ΦX174
HsaDRG1 protein

g)

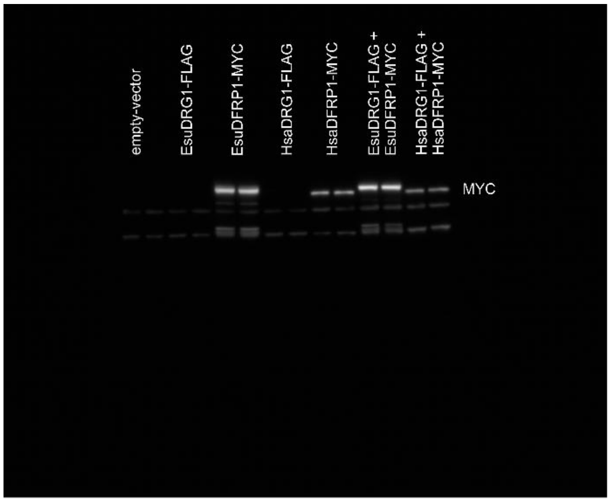
empty-vector
EsuDRG1-FLAG
EsuDFRP1-MYC
HsaDRG1-FLAG
HsaDFRP1-MYC
EsuDRG1-FLAG +
EsuDFRP1-MYC
HsaDRG1-FLAG +
HsaDFRP1-MYC
MYC

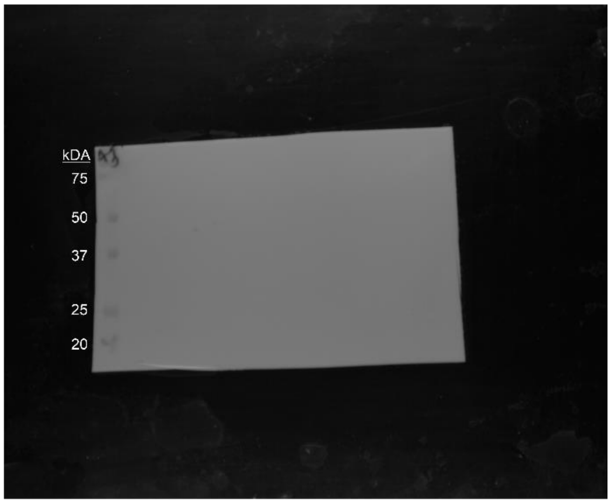
kDA
75
50
37
25
20

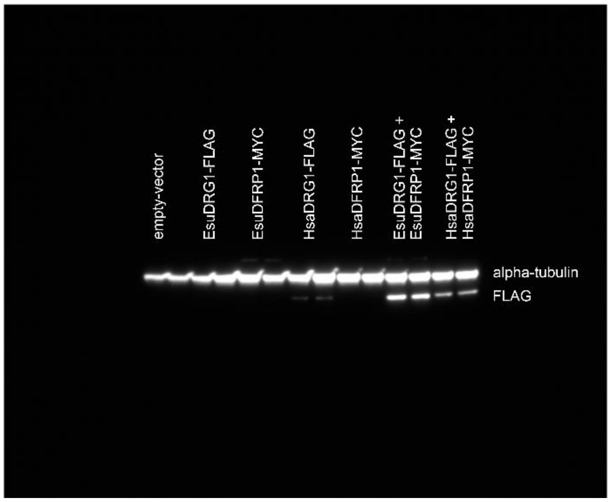
empty-vector
EsuDRG1-FLAG
EsuDFRP1-MYC
HsaDRG1-FLAG
HsaDFRP1-MYC
EsuDRG1-FLAG +
EsuDFRP1-MYC
HsaDRG1-FLAG +
HsaDFRP1-MYC
alpha-tubulin
FLAG

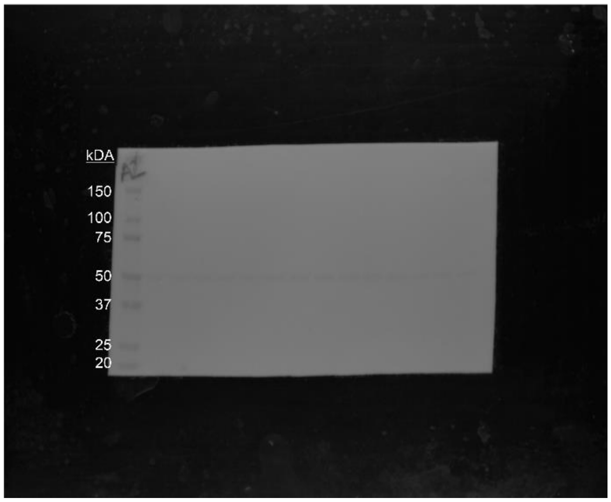
kDA
150
100
75
50
37
25
20

h)

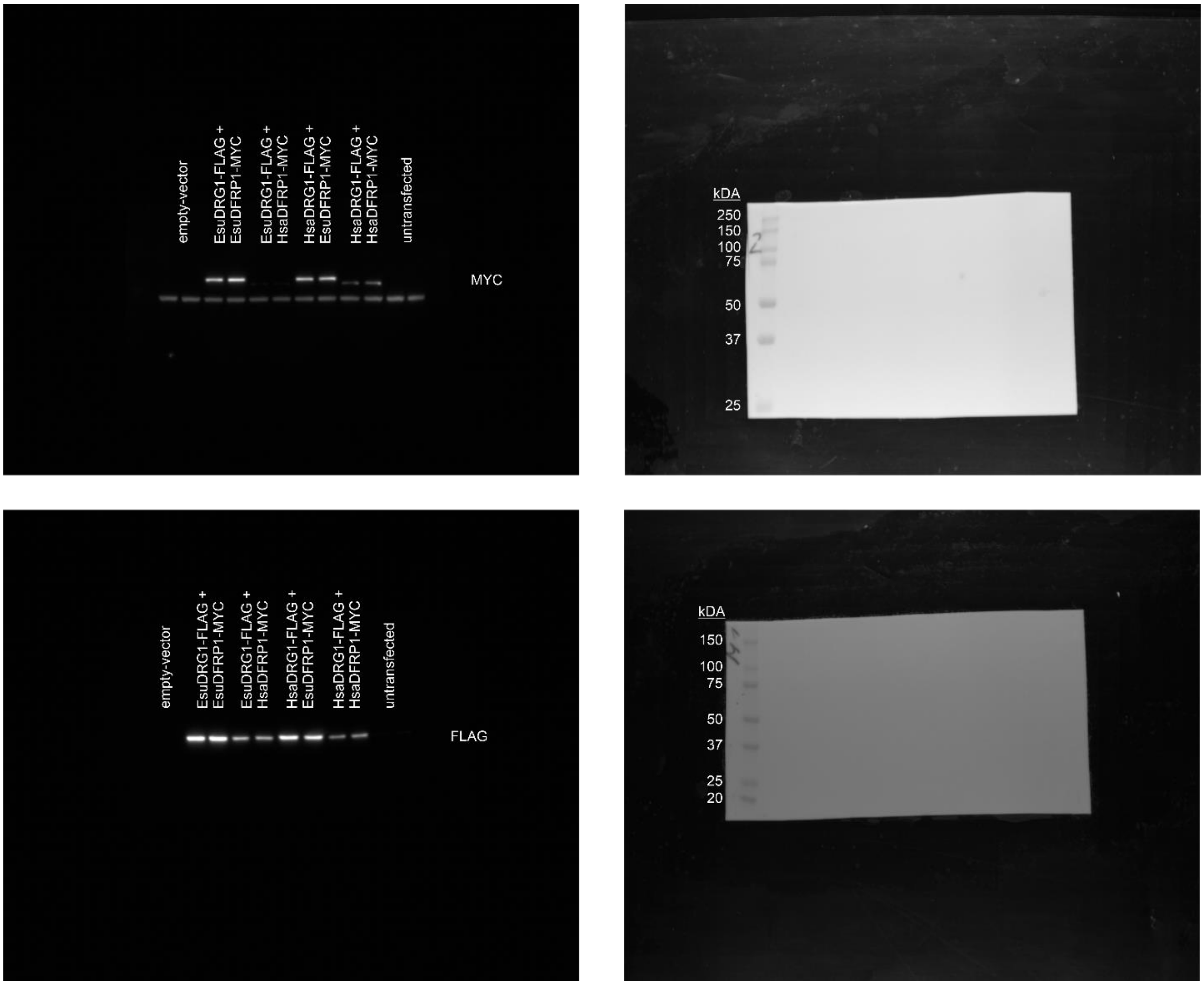
empty-vector
EsuDRG1-FLAG + EsuDFRP1-MYC
EsuDRG1-FLAG + HsaDFRP1-MYC
HsaDRG1-FLAG + EsuDFRP1-MYC
HsaDRG1-FLAG + HsaDFRP1-MYC
untransfected
MYC
kDA
250
150
100
75
50
37
25
empty-vector
EsuDRG1-FLAG + EsuDFRP1-MYC
EsuDRG1-FLAG + HsaDFRP1-MYC
HsaDRG1-FLAG + EsuDFRP1-MYC
HsaDRG1-FLAG + HsaDFRP1-MYC
untransfected
FLAG
kDA
150
100
75
50
37
25
20

i)

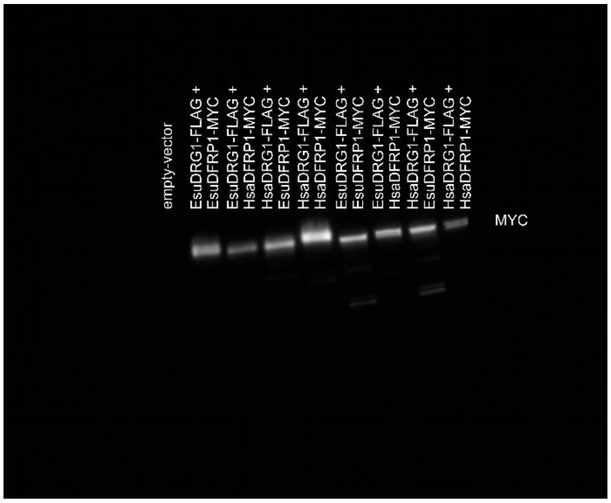
empty-vector
EsuDRG1-FLAG + EsuDFRP1-MYC
EsuDRG1-FLAG + HsaDFRP1-MYC
HsaDRG1-FLAG + EsuDFRP1-MYC
HsaDRG1-FLAG + HsaDFRP1-MYC
EsuDRG1-FLAG + EsuDFRP1-MYC
EsuDRG1-FLAG + HsaDFRP1-MYC
HsaDRG1-FLAG + EsuDFRP1-MYC
HsaDRG1-FLAG + HsaDFRP1-MYC
MYC

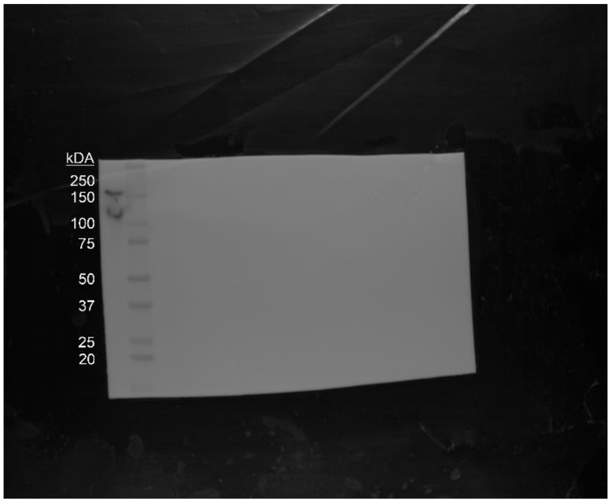
kDA
250
150
100
75
50
37
25
20

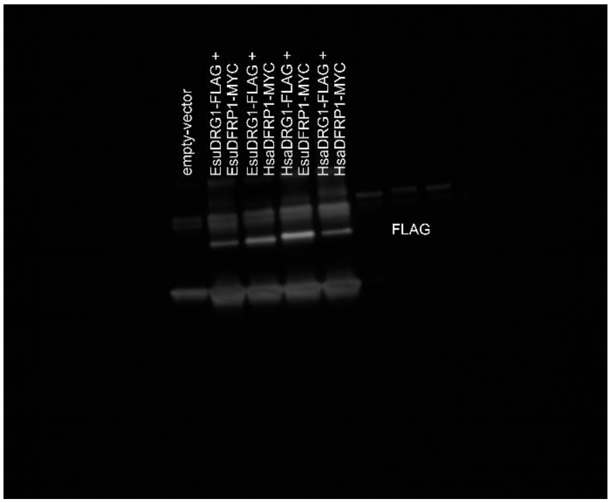
empty-vector
EsuDRG1-FLAG + EsuDFRP1-MYC
EsuDRG1-FLAG + HsaDFRP1-MYC
HsaDRG1-FLAG + EsuDFRP1-MYC
HsaDRG1-FLAG + HsaDFRP1-MYC
FLAG

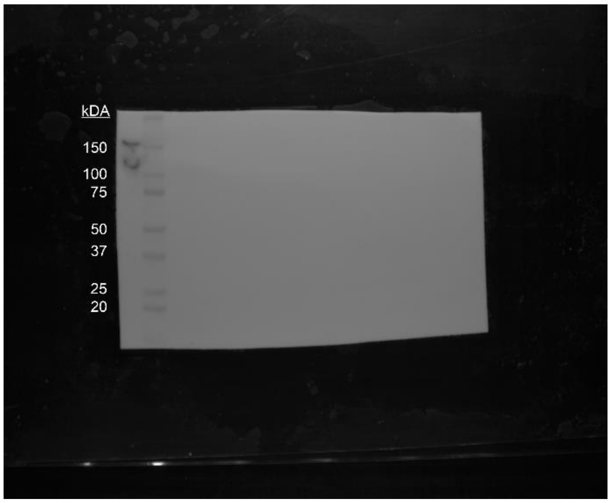
kDA
150
100
75
50
37
25
20

j)

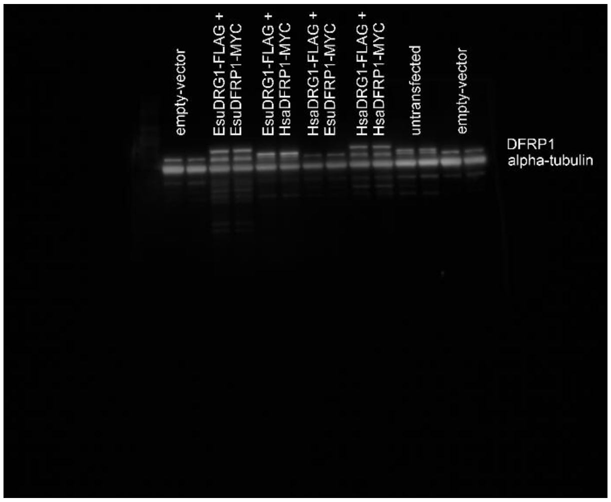
empty-vector
EsuDRG1-FLAG + EsuDFRP1-MYC
EsuDRG1-FLAG + HsaDFRP1-MYC
HsaDRG1-FLAG + EsuDFRP1-MYC
HsaDRG1-FLAG + HsaDFRP1-MYC
untransfected
empty-vector
DFRP1
alpha-tubulin

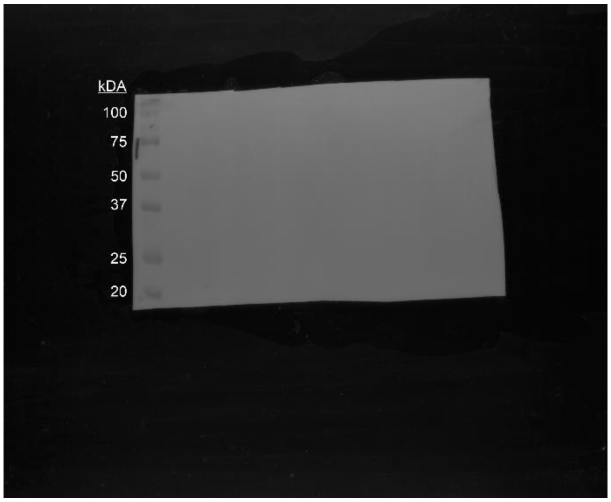
kDA
100
75
50
37
25
20

Fig. S10. Original images of gels and western blots before cropping.

a) Full-length gels in Fig. 4a,b.

b) Full-length blots in Fig. 4d.

c) Full-length gels in Fig. 5a.b.

d) Full-length gel in Fig. 5d.

e) Full-length blots in Fig. 5e.

f) Full-length gels in Fig. 8a,b,c,d,e.

g) Full-length blots in Fig. 9a.

h) Full-length blots in Fig. 9b.

i) Full-length blots in Fig. 9c.

j) Full-length blot in Fig. 10a.
